# An earth system model shows self-sustained melting of permafrost even if all man-made GHG emissions stop in 2020

**DOI:** 10.1038/s41598-020-75481-z

**Published:** 2020-11-12

**Authors:** Jorgen Randers, Ulrich Goluke

**Affiliations:** BI Norwegian Business School, Oslo, Norway

**Keywords:** Biogeochemistry, Climate sciences, Planetary science

## Abstract

The risk of points-of-no-return, which, once surpassed lock the world into new dynamics, have been discussed for decades. Recently, there have been warnings that some of these tipping points are coming closer and are too dangerous to be disregarded. In this paper we report that in the ESCIMO climate model the world is *already* past a point-of-no-return for global warming. In ESCIMO we observe self-sustained melting of the permafrost for hundreds of years, even if global society stops all emissions of man-made GHGs immediately. We encourage other model builders to explore our discovery in their (bigger) models, and report on their findings. The melting (in ESCIMO) is the result of a continuing self-sustained rise in the global temperature. This warming is the combined effect of three physical processes: (1) declining surface albedo (driven by melting of the Arctic ice cover), (2) increasing amounts of water vapour in the atmosphere (driven by higher temperatures), and (3) changes in the concentrations of the GHG in the atmosphere (driven by the absorption of CO_2_ in biomass and oceans, and emission of carbon (CH_4_ and CO_2_) from melting permafrost). This self-sustained, in the sense of no further GHG emissions, melting process (in ESCIMO) is a causally determined, physical process that evolves over time. It starts with the man-made warming up to the 1950s, leading to a rise in the amount of water vapour in the atmosphere—further lifting the temperature, causing increasing release of carbon from melting permafrost, and simultaneously a decline in the surface albedo as the ice and snow covers melts. To stop the self-sustained warming in ESCIMO, enormous amounts of CO_2_ have to be extracted from the atmosphere.

## Introduction

The possibility of points-of-no-return in the climate system has been discussed for two decades^1–3^. A point-of-no-return can be seen as a threshold which, once surpassed, fundamentally changes the dynamics of the climate system. For example, by triggering irreversible processes like melting of the permafrost, drying of the rainforests, or acidification of surface waters. Recently, Lenton et al.^4^ summarized the global situation and warned that thresholds may be closer in time than commonly believed.

The purpose of this article is to report that we have identified a point-of-no-return in our climate model ESCIMO—and that it is already behind us. ESCIMO is a “reduced complexity earth system” climate model^5^ which we run from 1850 to 2500. In ESCIMO the global temperature keeps rising to 2500 and beyond, *irrespective* of how fast humanity cuts the emissions of man-made greenhouse gas (GHG) emissions. The reason is a cycle of self-sustained melting of the permafrost (caused by methane release), lower surface albedo (caused by melting ice and snow) and higher atmospheric humidity (caused by higher temperatures). This cycle appears to be triggered by global warming of a mere + 0.5 °C above the pre-industrial level.

## Method

We used ESCIMO to simulate the development of the global climate system from 1850 to 2500 under different assumptions concerning the emission of man-made GHGes. ESCIMO is a system dynamics model that includes representations of the world’s atmosphere, oceans, forests (and other land types), biomass—and their interactions. It is described here^5^. The source code with documentation is available online^6^.

In the first simulation reported here, “Scenario 1”, we assume that humanity reduces man-made GHG emissions to zero by 2100. In the second simulation, “Scenario 2”, we assume that emissions are cut much faster—to zero in 2020. In both cases man-made emissions remain zero thereafter.

## Results

**Figure 1:**
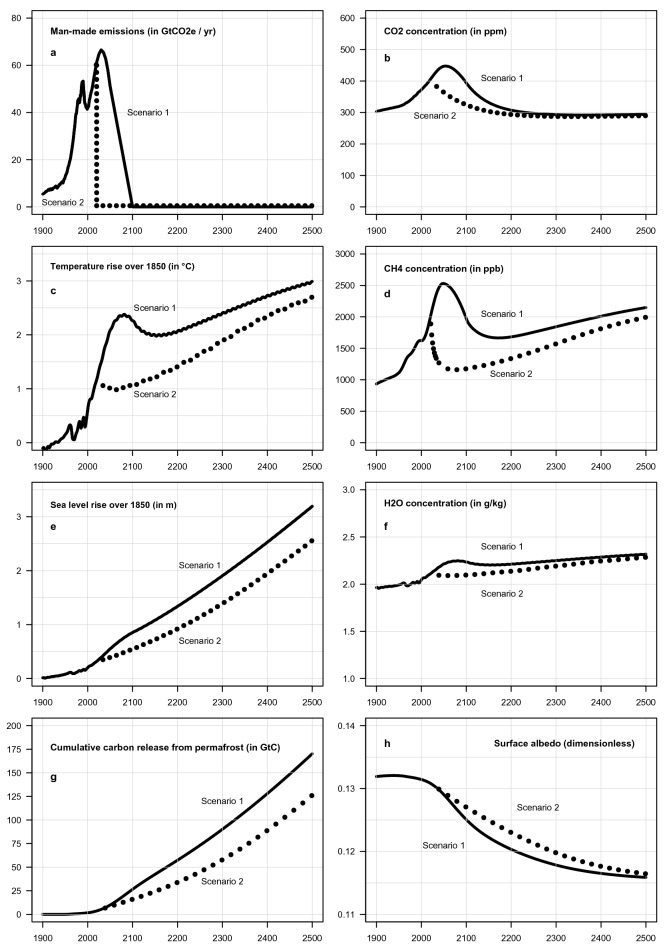
Man-made greenhouse gas (GHG) emissions (**a**), the global average surface temperature (**c**), sea level rise (**e**), and cumulative release of carbon from permafrost (**g**) in two scenarios from 1900 to 2500, generated by ESCIMO. Also shown are the concentration in the atmosphere of CO_2_ (**b**), CH_4_ (**d**), H_2_O (**f**), and surface albedo (**h**). Solid black curves show Scenario 1 where man-made GHG emissions are phased out by 2100. Black dotted curves show Scenario 2 where man-made GHG emissions are cut to zero in 2020. In both cases the global temperature keeps rising for hundreds of years after all man-made emissions have ceased.

The result is shown in Fig. 1. In both scenarios the global temperature keeps rising for hundreds of years—to around + 3 °C in 2500—after a temporary decline in this century in conjunction with the decline in man-made emissions (Fig. 1c). The sea level rises monotonically to around + 3 m in 2500 (Fig. 1e).

### Scenario 1

Scenario 1 describes the result when we assume that man-made emissions peak in the 2030s and decline to zero in 2100 (see Fig. 1, solid lines). This is the “most likely” scenario as described here^7^.

The historical part of the simulation (1850–2015) and the ensuing 60 years (2015–2075) are intuitive and understandable. Rising emissions of man-made GHGes lead to an increase in the concentration of GHGes in the atmosphere (Fig. 1b,d). This, in turn, leads to a rise in the global average surface temperature because GHG molecules block outgoing long-wave (heat) radiation from the surface. The warming is enhanced by the increased amount of water vapour which accumulates in a warmer atmosphere because H_2_O is a strong greenhouse gas which blocks other frequencies (Fig. 1f). The warming leads to rising sea levels because of thermal expansion and glacier run-off. Difficult to detect, but of great significance for the years beyond 2150, surface albedo starts a slow and smooth decline as the ice and snow cover melts, making the planet darker and leading to more absorption of short-wave (SW) radiation in the surface (Fig. 1h).

In Scenario 1 the temperature passes a temporary peak around 2075 at + 2.3 °C above pre-industrial times. The temperature then falls for 75 years (2075–2150) to + 2 °C. There are two reasons: (a) the concentration of GHGs in the atmosphere declines, and (b) heat is used to melt on-land glaciers and Arctic ice.

Furthermore, the concentration of CO_2_ declines (from its all-time peak of 450 ppm in 2050) through two processes: (a) CO_2_ is gradually absorbed in the ocean surface (and later transported into the deep ocean), and (b) CO_2_ is gradually absorbed in the biosphere. CO_2_ in the atmosphere is converted through photosynthesis into biomass in living matter and soils at a rate that is determined by the temperature and the amount of CO_2_ in the atmosphere. By 2150 all on-land snow and ice are gone in ESCIMO Scenario 1 (except in Greenland and Antarctica, which require thousands of years to melt).

While the developments to 2150 are understandable, developments in ESCIMO beyond 2150 are more surprising (counter-intuitive). As shown in Fig. 1 the temperature once more starts rising. The surprising fact is that this rise takes place 50 years *after* man-made emissions have ceased, and *after* the concentration of CO_2_ in the atmosphere has been significantly reduced through absorption in oceans and biomass.

**Figure 2:**
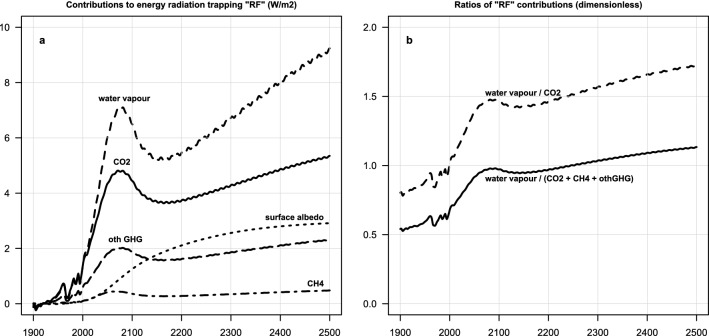
(**a**) The contribution to global warming (“energy radiation trapping”) from water vapour, CO_2_, CH_4,_ other GHGes, and surface albedo in Scenario 1. After 2150 the main drivers are water vapour and CO_2_, with albedo in the third place. The contribution of CH_4_ is much smaller, while the other Montreal and Kyoto gases remain the fourth most important driver of the self-sustained warming and melting of the permafrost. (**b**) The relative importance of water vapour in global warming in Scenario 1. After 2150 water vapor has approximately the same effect as the sum of all the other GHGes. Historically, from 1850 to 2000, the ratios in the ESCIMO base run fall well within the uncertainty band reported by Cess, Rind, Hansen and Ramanathan and Inamdar cited earlier.

The explanation (in ESCIMO) is as follows. While GHG concentrations—and thus their forcings—fall from 2070 to 2150, the effect of surface albedo continues on its smooth upward path throughout this period. Its time development is much slower than that of GHGes. It is the result of mainly Arctic ice melting—but it has enough ‘momentum’ to push the climate system back onto a path of rising temperatures, with its secondary effects of raising humidity and permafrost melting, which then in turn help the system become warmer and warmer, even if man-made GHG emissions are zero. A cycle of self-reinforcing processes is established. See Fig. 2a.

This cycle consists of decreasing surface albedo, increasing water vapour feedback and increasing melting of the permafrost, releasing carbon (both as CH_4_ and CO_2_), resulting in even further temperature rises, and so on. In a highly coupled feedback model like ESCIMO it is the chain of events, closing in on itself, that matters. Even after no more man-made GHG are emitted, this cycle/chain continues on its own. The process is self-sustaining, at least until all carbon is released from permafrost and all ice is melted.

### Scenario 2

Scenario 2 (see Fig. 1, dotted curves) was made to check whether humanity could avoid continuing warming from the self-sustained chain of circumstances of decreasing ocean albedo, increasing water vapour feedback and increasing melting of the permafrost by cutting man-made GHG emissions earlier than in Scenario 1. The answer is no. Figure 1 (dotted curves) shows that even if all man-made GHG emissions were (unrealistically) cut to zero in 2020, the temperature starts rising again after 2150—as a result of the cycle of self-sustaining processes of decreasing albedo, melting of the permafrost and increasing water vapour feedback.

## Discussion

### Unexpected result

The unexpected result in Scenarios 1 and 2 is that the global temperature keeps rising for centuries *after* man-made GHG emissions of are brought to zero. Even more surprising, at first glance, is the fact that the temperature keeps rising *after* the concentration of CO_2_ in the atmosphere has declined back to the pre-industrial level through absorption in the deep ocean, biomass and soil.

In both cases the explanation (in ESCIMO) rests in the joint action of albedo, carbon (both as CH_4_ and CO_2_) from melting permafrost, and water vapour in warm air—which together ensure that the temperature stays high even when the concentration of CO_2_ declines. Some additional comments help explain:

### The planet gets darker: the role of albedo

As temperature rises, ice and snow are melted, making the planet darker. Between 2070 and 2300, for example, the average *ocean* albedo (in ESCIMO) declines from 0.080 to 0.067, and the *surface* albedo from 0.127 to 0.117, see Supplement Figure 15. As a result, more short-wave radiation is absorbed. In ESCIMO about 1.7 Wm^−2^—which is enough to trigger and drive significant change in the delicately balanced, global climate system.

### Water vapour feedback

Water vapour exists in the atmosphere because of the balance between evaporation, which increases with temperature, and precipitation from the atmosphere, which also increases with temperature. H_2_O is *not* held in the atmosphere because of CO_2_, or any other GHG. This means that water vapour, and its warming effect, will *not* disappear when the CO_2_ concentration declines back to pre-industrial levels—as long as the temperature stays high enough.

### Comparing the effect of GHGs, albedo and water vapour on the energy balance of earth

The relative importance of albedo, water vapour, and release of carbon from permafrost over time can be illustrated in terms of the “radiative forcing” each contributes. There are two ways to estimate radiative forcings, one, using the IPCC^8^ formulas for GHGs, and two, deducing the radiative forcing from changes in the long-wave radiation back to space (LW-ToA). The first works well, at least historically, for CO_2_, CH_4_, N_2_O and the other greenhouse gases (We show the result of calculating GHG radiative forcing in ESCIMO using the IPCC formulas in Supplement Figure 16), but not for climate feedbacks like water vapour and albedo. For water vapour, “radiative forcing” is generally not used (IPCC^8^, pg. 666), because it modifies the forcing of other forcing agents (Ramanathan and Inamdar^9^, pg. 121). Instead, using the effectiveness in absorbing thermal radiation of GHGs, including H_2_O and albedo, is an acceptable proxy for estimating their “radiative forcing”. This approach was used by Hansen et al.^10^, Rind^11^ and Ramanathan and Inamdar^9^, who built on the conceptual work of Cess^12^.

Figure 2a compares the “radiative forcings”, defined as G_a_, i.e. the difference between LWToA clear sky radiation and LW cloudy ToA radiation, normalized to 1850 (for detail, see Rind^11^, pg. 260) of CO_2_, CH_4_, other GHGes, water vapour and surface albedo. “The observed value of G_a_ = 146 Wm^−2^ K^−1^; clouds increase the value by about 33 Wm^−2^ K^−1^ (Raval and Ramanathan^13^).” The values for ESCIMO in 1995 are G_a_ = 148 Wm^−2^ K^−1^; clouds increase the value by about 30 Wm^−2^ K^−1^.

Surface albedo affects the SW radiation balance at the surface. Thus, to estimate the “radiative forcing” of surface albedo, we follow a similar logic as for water vapour: we compare the SW reflection of the surface at time_t_ to the SW reflection of the surface at time_1850_. The surface albedos are shown in Supplement Figure 15. Land albedo in ESCIMO rises ever so slightly in historical times, recreating the negative LUC “forcing” reported by IPCC. The ocean albedo in ESCIMO drops, because of the melting of the arctic ice.

The declining albedo leads to an increase in the amount of short-wave radiation absorbed in the surface. The “radiative forcing of delta albedo” in the ESCIMO base run is shown in Fig. 2a dotted curve. Numerically, it rises from 0.8 Wm^−2^ in 2070 to 2.6 Wm^−2^ in 2300 in the ESCIMO base run, an increase of 1.7 Wm^−2^.

Thus, the drop in albedo is the trigger of the resumed self-sustained melting of permafrost after 2150, aided in the real world, and in ESCIMO, by water vapour and the consequent continued release of carbon from the melting permafrost.

### Match with other models

We have compared ESCIMO with other models, with particular focus on the assumptions that are driving the self-sustained melting of the permafrost. In ESCIMO we assume that the permafrost melts as a consequence of the transfer of heat from the atmosphere to the frozen soil. We assume that the rate of heat transfer is proportional to the temperature difference between air and frozen soil. Furthermore, we assume that the resulting tundra, after some delay, will start absorbing CO_2_ through photosynthesis once plants start establishing themselves on the formerly frozen ground. The absorption rate depends on the temperature and the concentration of CO_2_ in the atmosphere (CO_2_ stimulates plant growth). Needless to say, the causal mechanisms included in ESCIMO are very aggregate and far from a detailed description of the complex melting process in the real permafrost.

In order to check whether our assumptions lead to reasonable results, we compared the output from ESCIMO with the output from other models, as reported in McGuire et al.^14^. The comparison is halting, since the temperature path in Scenario 1 differs from the path in the RCP4.5 scenario, which the other models use. We found that ESCIMO Scenario 1 generates a melting of 2 million km^2^ of permafrost by 2300, compared to 3–5 in other models. And that ESCIMO Scenario 1 releases an accumulated 175 billion tons of carbon (GtC), all from melting permafrost, by 2300, compared to plus 66–minus 70 in other models. Sadly, ESCIMO is not sufficiently regionalized to generate numbers for the amount of carbon which is absorbed in the vegetation that forms on the formerly frozen ground (which is 8–244 GtC in other models). ESCIMO only gives numbers for the extra carbon absorbed in *all* tundra, which, in ESCIMO, does not overlap one-to-one with formerly frozen ground, both old and new, which is 200 GtC. The uptake is due to accelerated humus formation fuelled by increased carbon uptake in the biomass of tundra during the period of high CO_2_ concentration. The comparison with other models seems to indicate that ESCIMO in Scenario 1 releases more carbon than other models, but it needs further investigation to decide whether this is because the RCP4.5 scenario differs from Scenario 1 in the centuries beyond 2100.

### Sensitivity analysis

We did a conventional sensitivity analysis to verify that the self-sustained melting of the permafrost is a robust phenomenon in ESCIMO—in other words, we checked that the continuing rise in the global average temperature does not depend on a very specific choice of the parameter values that determine the strength of the various processes in the model system. There are many (ca 100) such parameters in ESCIMO. They all have independent physical meaning, and each got a numerical value based on information from the literature. To do the sensitivity analysis, we first randomly picked 14 uncertain parameters from the model. Next, we independently sampled all 14 parameters from random uniform distributions with ranges of plus minus 10% around their standard value for 200 sensitivity runs.

**Figure 3:**
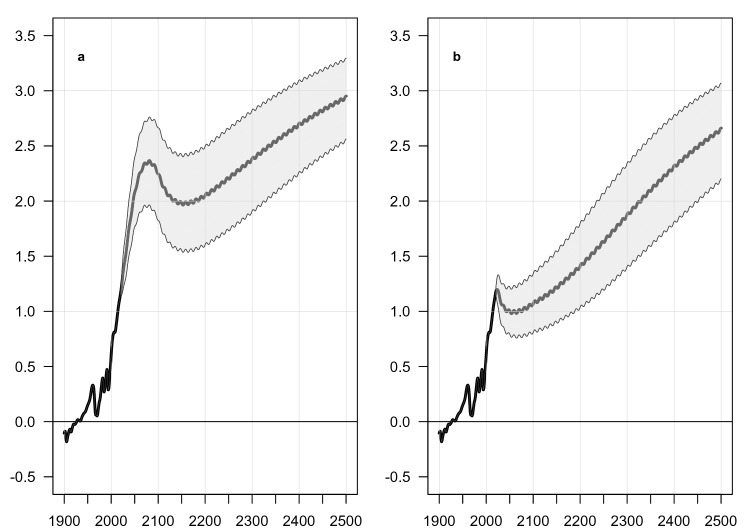
Sensitivity of the global average temperature to variation in parameter values in ESCIMO, for Scenarios 1 and 2. Sensitivity analysis of 14 randomly chosen uncertain parameters from the model. Sampled independently using Latin-Hypercube sampling from random uniform distributions with ranges of plus minus 10% around their standard value for 200 sensitivity runs. For the parameters see Supplement Table 1. Graph to the left shows Scenario 1 where man-made GHG emissions are phased out by 2100. Graph to the right shows Scenario 2 where man-made GHG emissions are cut to zero in 2020. Parameter variation does change absolute values but does not eliminate the broad pattern of self-sustained melting of the permafrost. The thick curve in the centre of the shaded area is the mean of the 200 runs. The shaded area covers 75% of all runs.

Figure 3 shows the result, for Scenarios 1 (a) and 2 (b). The grey band includes 75% of the 200 runs, for the central variable in ESCIMO, namely the temperature increase relative to 1850.

Our conclusion is that parameter variation has a strong effect on the absolute level of the future temperature in ESCIMO. But much more important, Fig. 3 shows that moderate variation in parameter values *does not* remove the self-sustained melting of the permafrost. The impact of the sensitivity experiment on five additional variables is shown in Supplement Figures 1 and 2. This broad pattern of development (in system dynamics language: this behaviour mode) remains the same. This is consistent with the system dynamics literature, which argues that it is normally not possible to predict future events in complex systems, while it is possible to say something meaningful about future dynamics (future behaviour modes).

It is, of course, simple to come up with parameter changes that remove the self-sustained melting of the permafrost—especially if those changes are made in what we already know are the most sensitive parts of ESCIMO, namely the equations that describe water vapour, albedo and clouds. But much more important, it is not simple to find parameter combinations that do so, while still being able to recreate the observed history from 1850 to 2015, as the standard parameter set in ESCIMO does.

We also did some further sensitivity analyses with parameters of special relevance for the study of permafrost melting, as described below.

### Further experiments to explore what it takes to stop the self-sustained melting of permafrost

**Figure 4:**
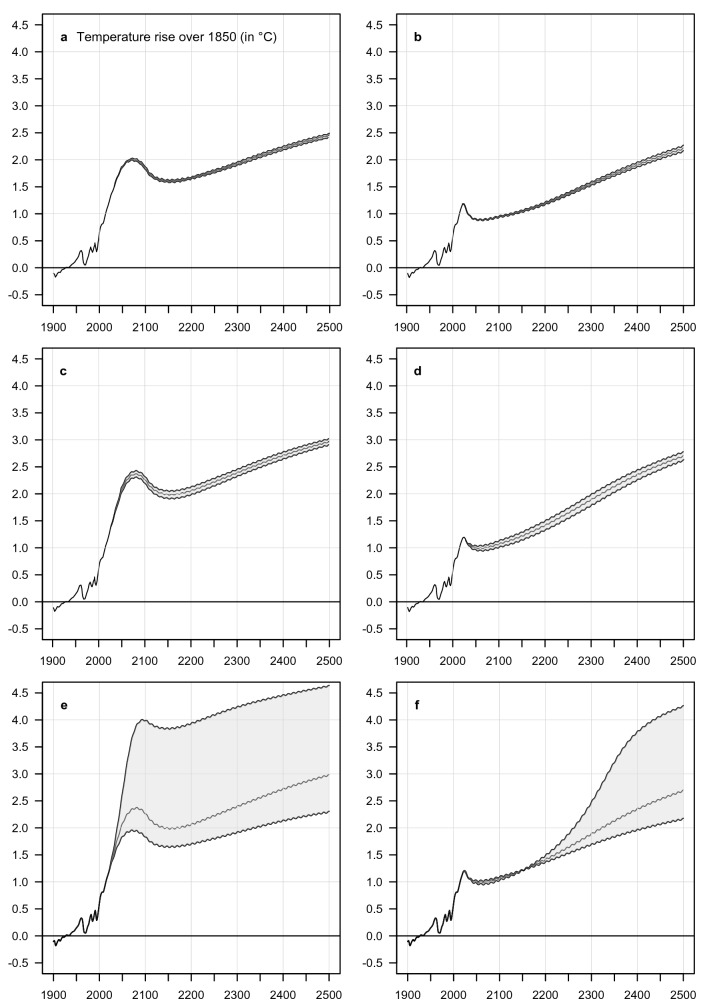
Sensitivity of the global average temperature to changes in three parameters that are central in permafrost melting. (1) The fraction of carbon that is converted (by bacteria) from CH_4_ to CO_2_ before it leaves the melting permafrost. (**a**,**b**) The shaded area includes 75% of the resulting runs. (2) The slope of the rate of melting of the permafrost that results from a given temperature. (**c**,**d**) The shaded area includes 75% of the resulting runs. (3) The slope of the future relationship between additional blocking of outgoing radiation and additional water vapour in the atmosphere—for values of humidity beyond what has been observed this far. (**e**,**f**) The detail about how we change the slope is given in Supplement Fiure 10.

We did further experiments with parameter variation, in order to study the robustness of the self-sustained melting of the permafrost. We chose to vary three parameters that have significant influence on the self-sustained melting process in ESCIMO. In Fig. 4 we show the effect of varying these three parameters. Our conclusion is that the effect on absolute rate of permafrost melting is significant, but that the *pattern* of self-sustained warming persists.

Figure 4 shows the effect on the global temperature, Supplement Figures 3–9 and 11–12 show the effects of each experiment for each scenario on five additional variables. Supplement Figure 10 gives details about the effect of changing the relationship between blocking and the amount of H_2_O in the atmosphere.

Our experiments involved the following three parameters:

1. *The fraction of carbon that is converted (by bacteria) from CH*_*4*_* to CO*_*2*_* before it leaves the melting permafrost.*
  The fraction of carbon that is released as CH_4_ in the real world is still unknown (Schneider von Deimling et al.^15^, Turetsky et al.^16^), so we explored the entire physically possible range from 0 (i.e. all carbon released from permafrost is released as CO_2_) to 1 (i.e. all carbon released from permafrost is released as CH_4_). See Fig. 4a,b and Supplement Figures 3 and 4. Since Lawrence et al.^17^ report a fraction between 2.5% for dry soil and 12% for wet soil we also re-ran our sensitivity analysis bounded by 0 and 15% to highlight this restricted range. See Supplement Figures 5, 6 and 7. Our choice of all carbon released from permafrost melting being released as methane for our base case results from the fact that we originally worked on ESCIMO between 2000 and 2015. At the time, we assumed, falsely as it turns out, that all carbon released from melting permafrost is released as methane. As reported above, in this paper we run sensitivity analysis where we changed the fraction of carbon released as methane from 0 to 100%, i.e. the entire physically possible range. And we run sensitivity analysis where we changed the fraction of carbon released as methane from 0 to 15%, i.e. around the currently assumed value of around 10%. In all the runs, the characteristic behaviour of self-sustained (in the sense of no more man-made GHG emissions) temperature rise is maintained.
2. *The slope of the rate of melting of the permafrost that results from a given temperature*.
  In ESCIMO, the rate of melting permafrost is measured in km^2^ per year. At our chosen reference temperature (4 °C) we assume that 12.500 km^2^ per year is melted to all depth^18,19,20^. To calculate the rate of melting at other temperatures we multiply with the following linear relationship:
  $$\begin{aligned}Effect \; of \; temperature\; (dimensionless) & = 1 + slope\; (dimensionless) \times {(Global \;average \;surface \;temperature}_{t} (^\circ \text{C}) \\ & \quad \div {Global \; average \;surface\; temperature}_{1850}(^\circ \text{C}) - 1)\end{aligned}$$
  See Fig. 4c,d and Supplement Figures 8 and 9.
3. *The slope of the future relationship between additional blocking of outgoing radiation as a function of additional water vapour in the atmosphere—for values of humidity beyond what has been observed this far*.
  To make this extrapolation we use the following 3rd order polynomial function:
  $$Fraction \; blocked =-0.2842*humidit{y}^{3}+1.8244*humidit{y}^{2}-3.7148*humiditiy+2.4523$$
  where the unit for humidity is g/kg. The historical part of the relationship has been calibrated to actual global average temperature. For detail, see Supplement Figure 10.
  See Fig. 4e,f and Supplement Figures 11 and 12.

### Summary of the three parameter changes

The chosen parameter variations do impact the *rate* of self-sustained melting of permafrost and its feedback effect on global temperature. But they do not *stop* the self-sustained melting of permafrost.

We believe that the reason for these results in ESCIMO is the importance of surface albedo rise, H_2_O blocking of long wave radiation and methane release from permafrost. As Fig. 2 above shows, we are seeing in ESCIMO a regime change from man-made emission driven warming until about 2200 to albedo and water-vapour driven warming beyond 2150.

### Remedial action

We did experiments with ESCIMO (see Supplement Figure 13) to explore (contra-factually) in what year man-made emissions must stop to avoid self-reinforcing melting of the permafrost. The answer is that all man-made emissions would have had to be cut to zero sometime between 1960 and 1970—when global warming was still below some + 0.5 °C.

Finally, we explored another strategy to stop self-sustained melting. We asked how much CO_2_ humanity must remove from the atmosphere every year from 2020 in order to avoid self-sustained temperature rise in the centuries ahead. The answer, in ESCIMO, proved to be at least 33 GtCO_2_e per year, for example through direct CO_2_ capture or biomass CCS (see Supplement Figure 14 (a) and (b)). In other words, building 33.000 big CCS plants and keep them running for ever.

This is technically feasible but would be hugely expensive. Cheaper opportunities exist to stop self-sustained global warming (through various forms of geo-engineering), but these will have unintended and undesired side effects beyond lowering the temperature.

## Conclusion

Self-sustained melting of the permafrost is a robust phenomenon in ESCIMO. It only disappears when man-made emissions are stopped counterfactually as early as in the 1960es. Or by choosing parameter values that do not recreate historical developments. We encourage other model builders to explore these conclusions in their models, and report on their findings.

## Supplementary information

Supplementary Information.
