# Author Correction: An earth system model shows self-sustained thawing of permafrost even if all man-made GHG emissions stop in 2020

**DOI:** 10.1038/s41598-021-81695-6

**Published:** 2021-02-02

**Authors:** Jorgen Randers, Ulrich Goluke

**Affiliations:** BI Norwegian Business School, Oslo, Norway

Correction to: *Scientific Reports* 10.1038/s41598-020-75481-z, published online 12 November 2020

The Authors now recognise that the acronym for the model used in the Article and originally reported in^1^ is culturally insensitive and inappropriate. The Authors did not intend to insult any ethnic groups by using the acronym for this software model.

Additionally, the original version of the Article contained an error. The text referred to permafrost melting where it should refer to permafrost thawing (which is a correct name for this process). This term has been corrected in the title, the abstract, throughout the text of the Article, and in the Supplementary Information file.

These changes do not affect the overall conclusions of the study. The errors have now been corrected in the PDF and HTML versions of the Article.

## References

[1] Randers J, Golüke U, Wenstøp F, Wenstøp S (2016). A user-friendly earth system model of low complexity: The ESCIMO system dynamics model of global warming towards 2100. Earth Syst. Dynam..

