# Supplementary material for: An earth system model shows self-sustained melting of permafrost even if all man-made GHG emissions stop in 2020
Source: Sci Rep. 2020 Nov 12;10:18456. doi: 10.1038/s41598-020-75481-z (PMC7661724; doi:10.1038/s41598-020-75481-z)
Supplement: Supplementary file 1 — Supplementary Information. [file 41598_2020_75481_MOESM1_ESM.pdf]

Supplementary material for

“An earth system model shows self-sustained melting of permafrost even if all man-made GHG emissions stop in 2020.”

Jorgen Randers*
Ulrich Goluke*

* BI Norwegian Business School

Version Sept. 20, 2020

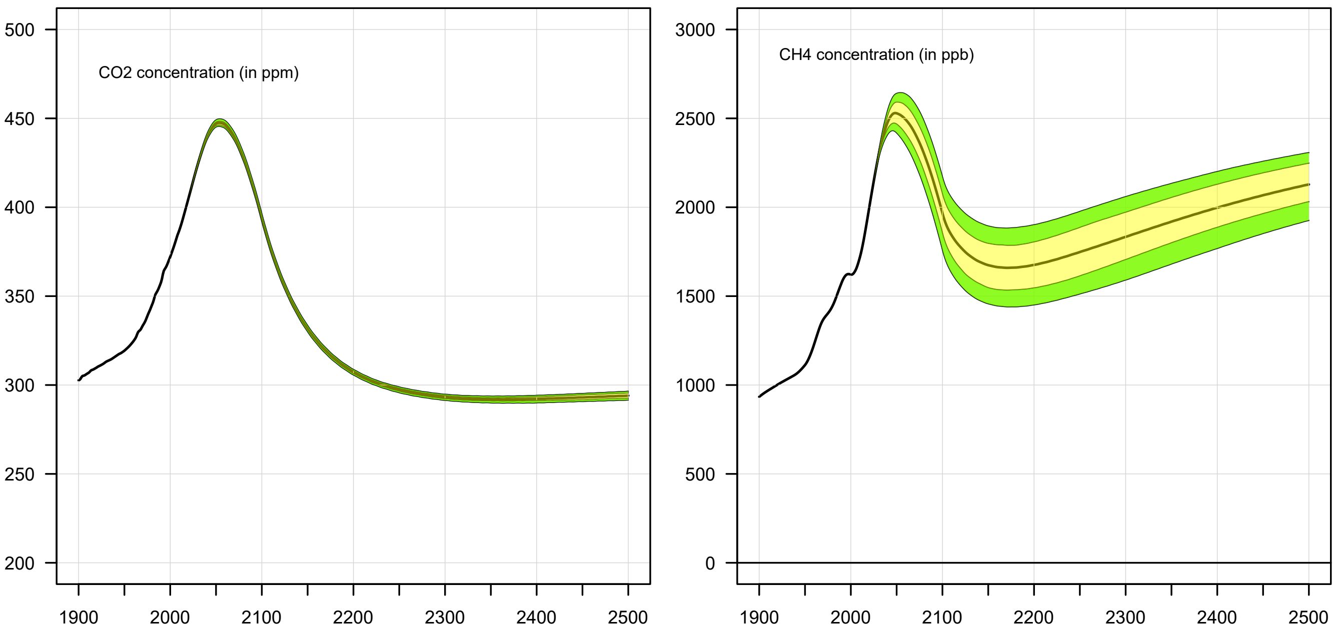

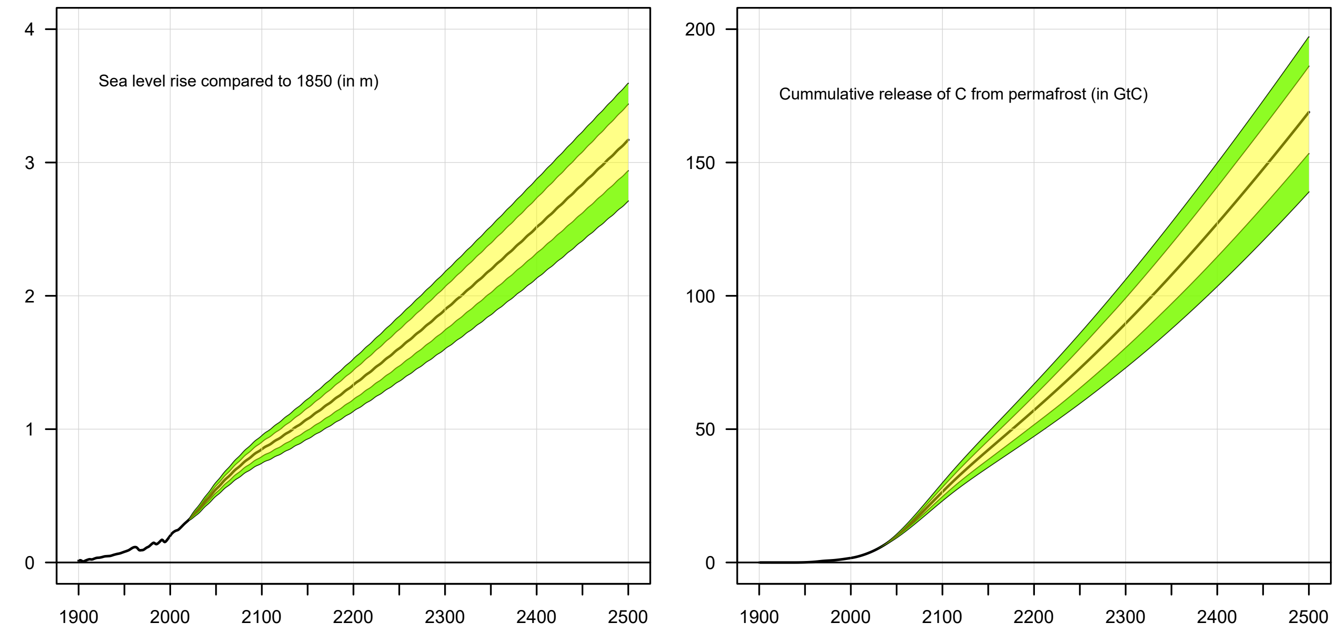

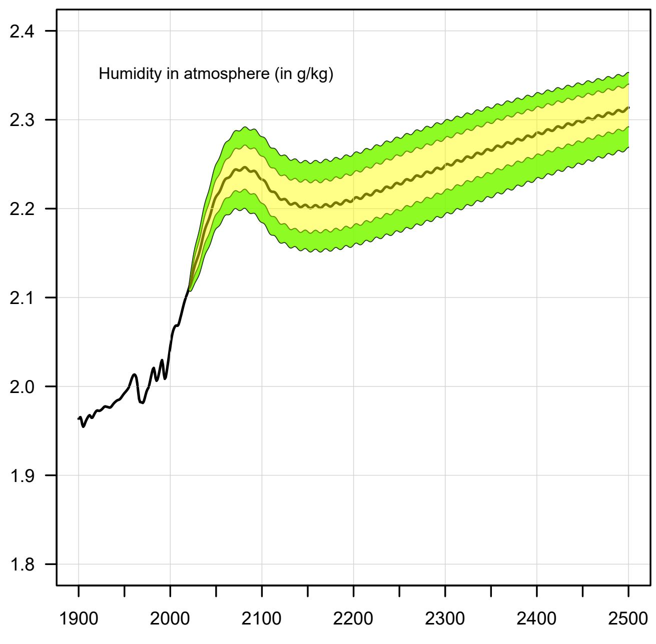

Supplement Figure 1: For scenario 1

Sensitivity analysis of 14 randomly chosen uncertain parameters from the model. Sampled independently using Latin-Hypercube sampling from random uniform distributions with ranges of plus minus 10 % around their standard value for 200 sensitivity runs. For the parameters see Supplement Table 1.

In *all* runs, the self-sustaining melting of permafrost is maintained in the model.

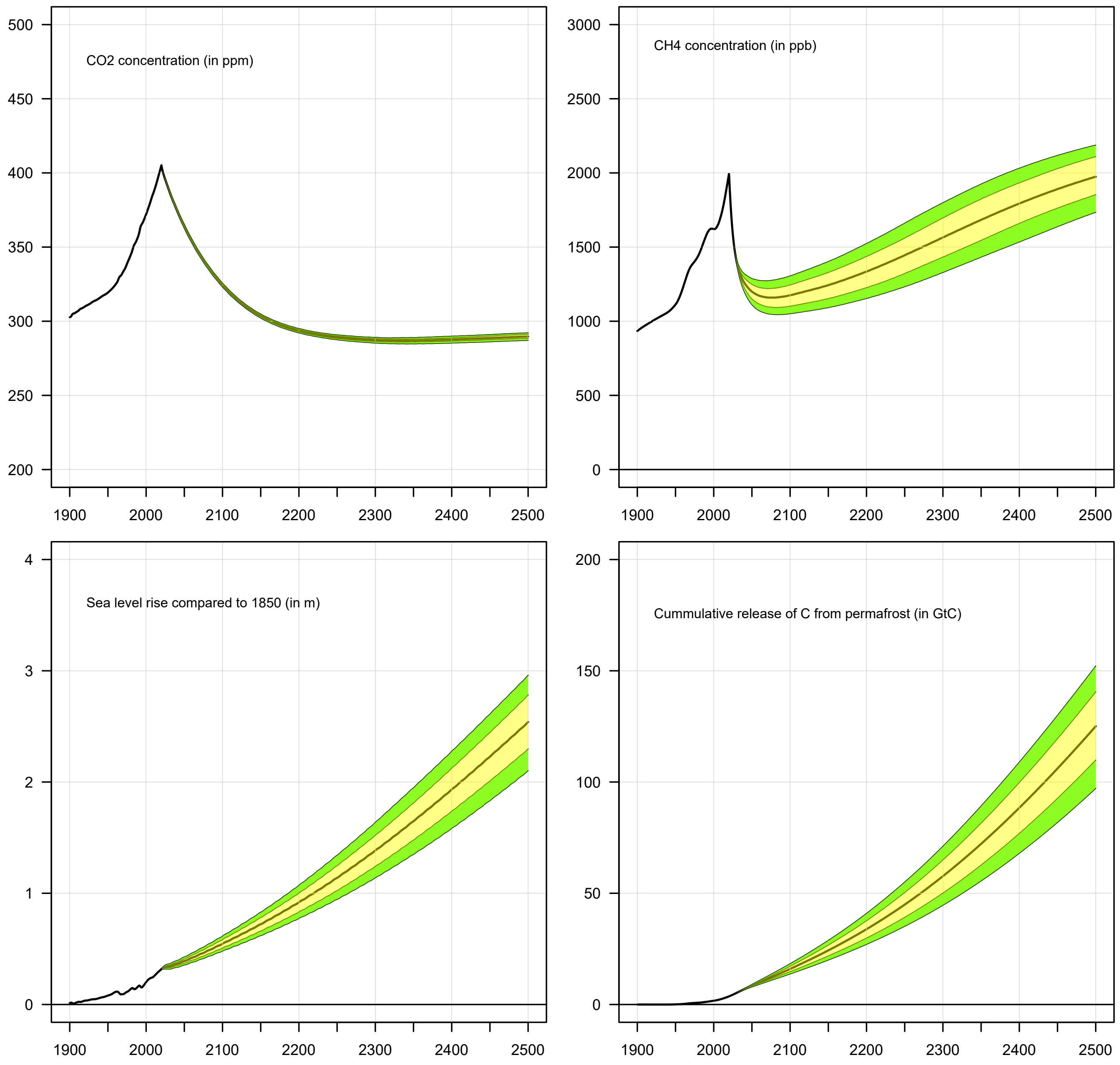

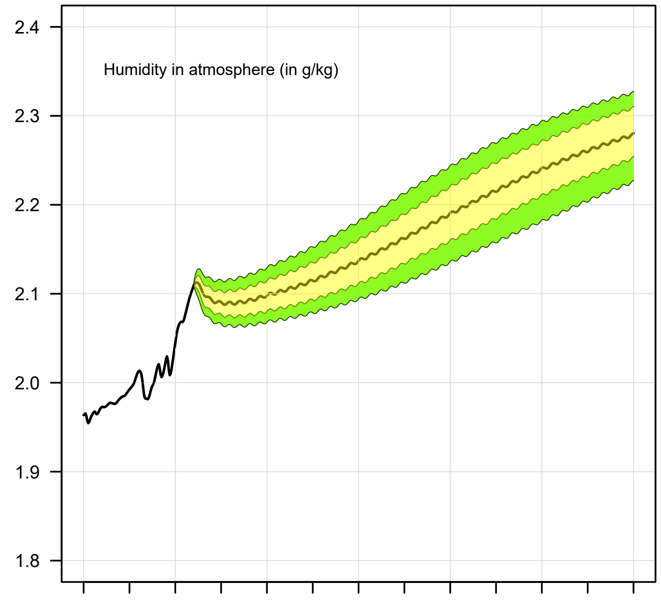

Supplement Figure 2: For scenario 2

Sensitivity analysis of 14 randomly chosen uncertain parameters from the model. Sampled independently using Latin-Hypercube sampling from random uniform distributions with ranges of plus minus 10 % around their standard value for 200 sensitivity runs. For the parameters see Supplement Table 1.

In *all* runs, the self-sustaining melting of permafrost is maintained in the model.

Supplement Table 1

The parameters for the sensitivity analysis of 14 randomly picked uncertain parameters from the model. Sampled independently using Latin-Hypercube sampling from random uniform distributions with ranges of plus and minus 10 % around their standard value for 200 sensitivity runs. Results for **Scenario 1** are shown in Figure 3a and Supplement Figure 1, results for **Scenario 2** are shown in Figure 3b and Supplement Figure 2.

Variable	Units	Standard value*	Plus 10%	Minus 10%
Northern Forest average lifetime of biomass	year	60	54	66
Northern Forest speed of regrowth	year	3	2.7	3.3
Slope of effect of temperature of shifting DESERT biome area to GRASS biome area	dimensionless	0.4	0.36	0.44
Slope of effect of temperature of glacial ice melting	dimensionless	1	0.9	1.1
Average time of water in ocean downward trunk	year	235	211	258
Albedo Antarctic	dimensionless	0.7	0.63	0.77
Time to degrade Kyoto gases in atmosphere	year	50	45	55
Time to regrow Northern Forests after fire	year	30	27	33
TROPical runoff time of carbon in soil to ocean	year	2000	1800	2200
TROPical time to decompose undisturbed dead biomass	year	24	21.6	26.4
Net heat flow between surface and deep ocean per °K of difference	ZetaJoules per year per °Kelvin	10	9	11
Albedo glacier	dimension-less	0.4	0.36	0.44
Average thickness glaciers	km	0.23	0.207	0.253
Average thickness Antarctic ice	km	2.14	1.926	2.354

* Reasons for the standard values are given in the documentation of the model, available here: http://www.2052.info/escimo/

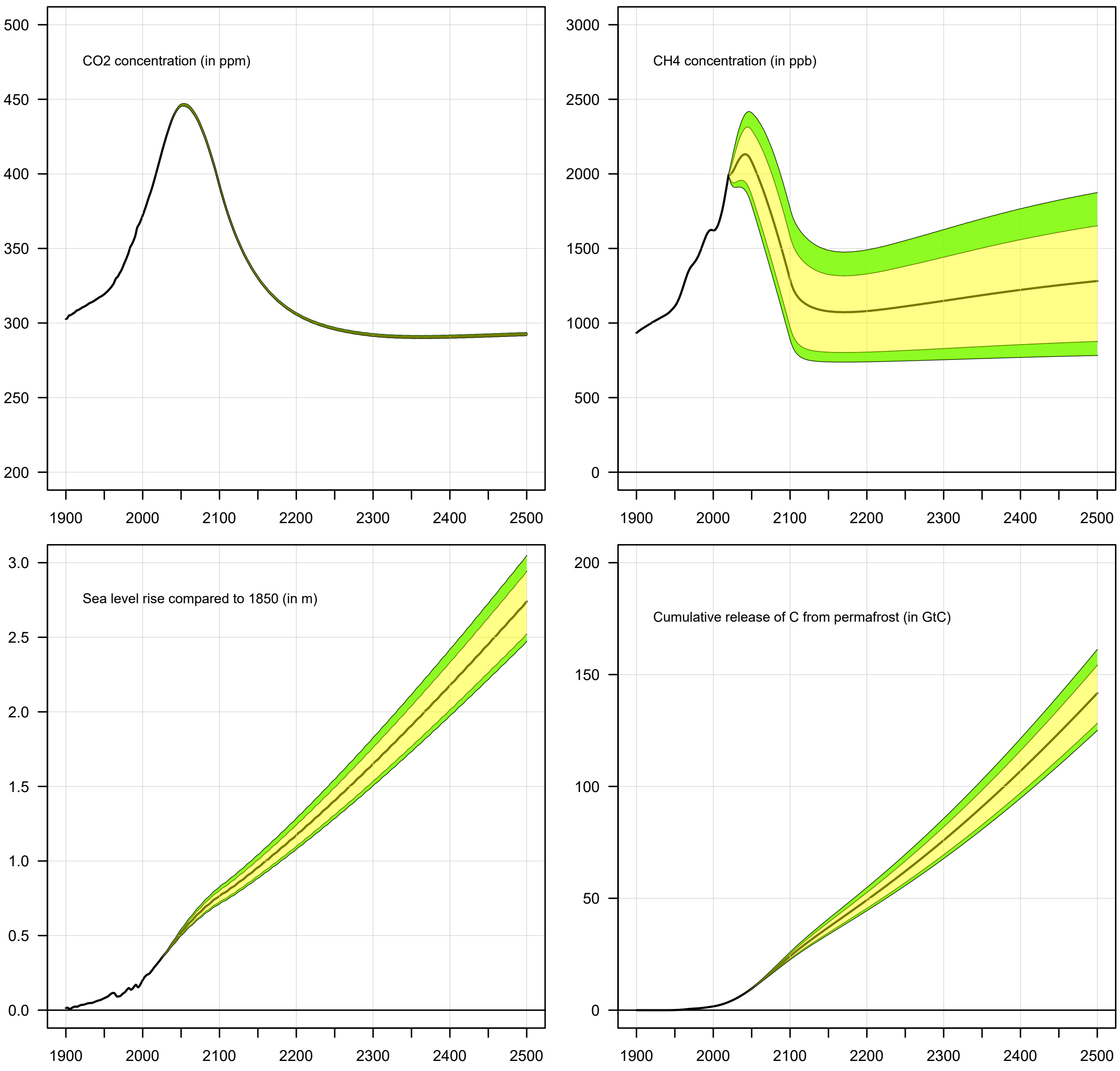

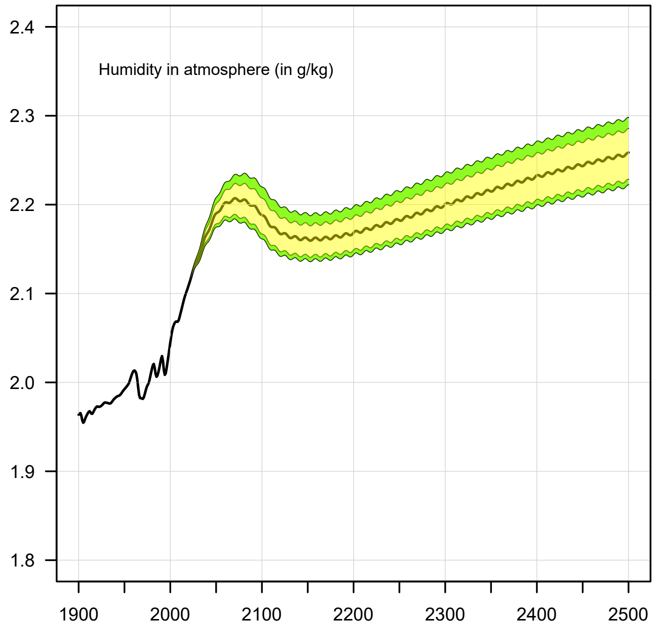

Supplement Figure 3: For scenario 1

Sensitivity to change in the fraction of carbon that is converted (by bacteria) from CH_4 to CO_2 before it leaves the melting permafrost. In the base run of ESCIMO all C being released from melting permafrost is released as CH_4. In these runs we uniformly sample this fraction from 0% (all C released as CO_2) to 100% (all C released as CH_4) for 500 runs. The yellow area in each graph shows 50% of the resulting runs, the green area extends this to show 75% of the resulting runs.

In *all* runs, the self-sustaining melting of permafrost is maintained in the model.

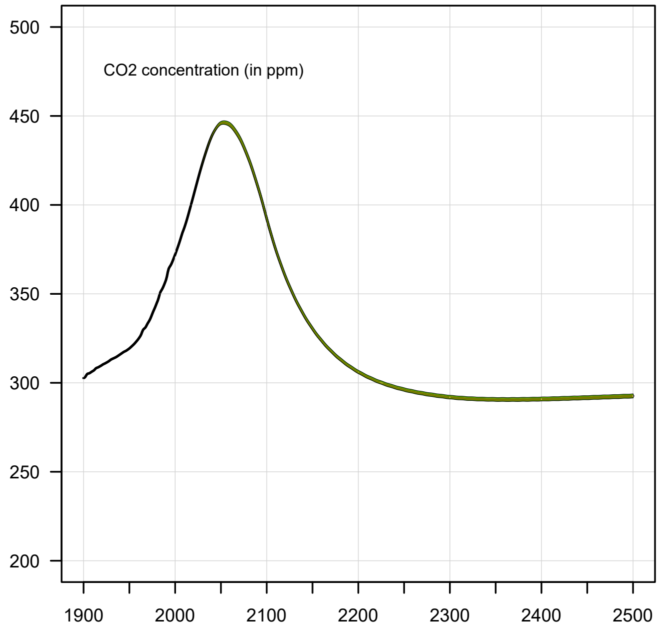

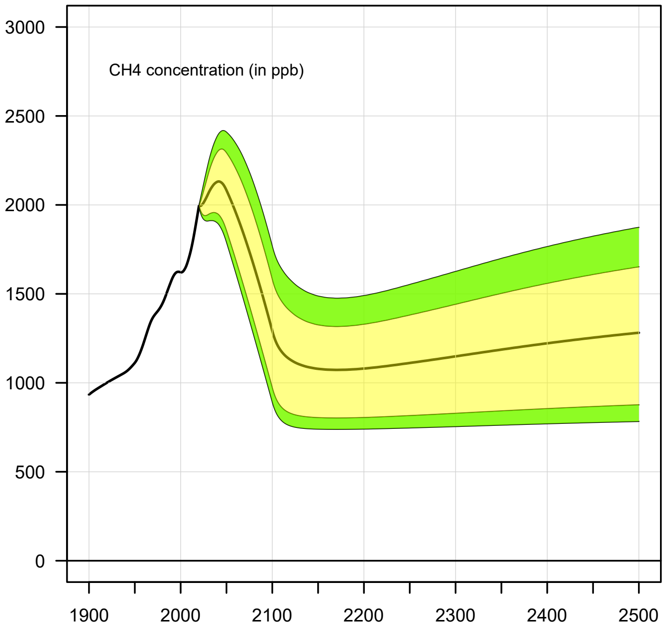

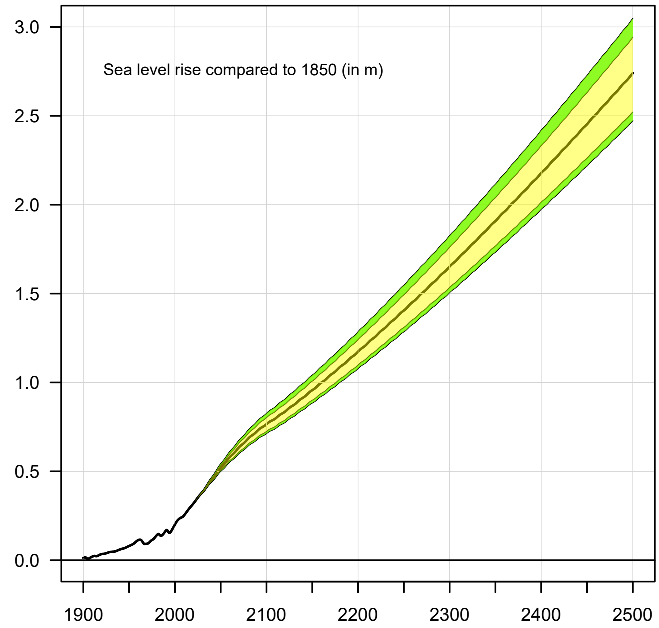

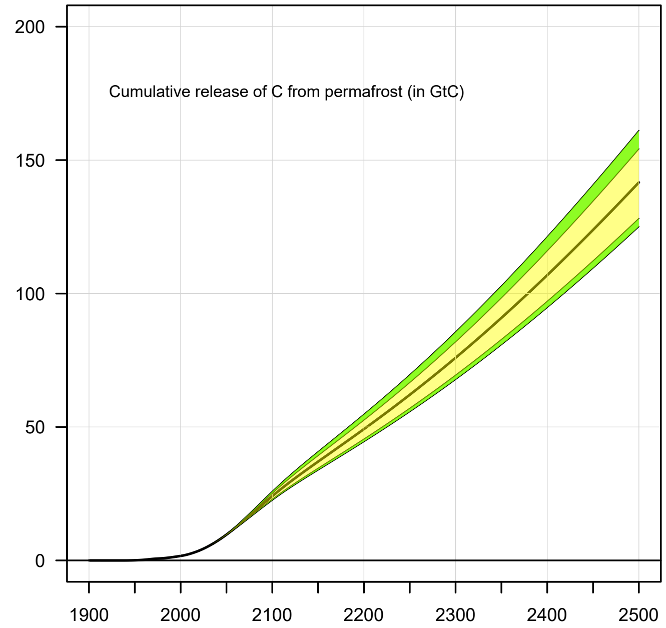

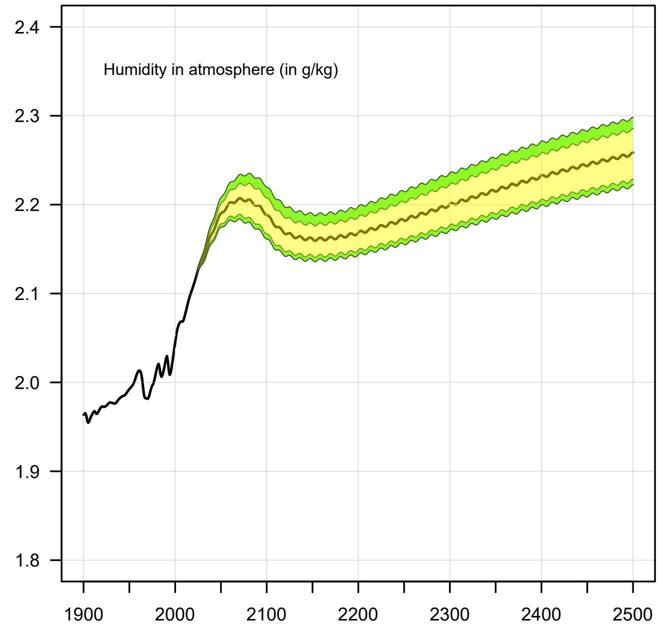

Supplement Figure 4: For scenario 2

Sensitivity to change in the fraction of carbon that is converted (by bacteria) from CH_4 to CO_2 before it leaves the melting permafrost. In the base run of ESCIMO all C being released from melting permafrost is released as CH_4. In these runs we uniformly sample this fraction from 0% (all C released as CO_2) to 100% (all C released as CH_4) for 500 runs. The yellow area in each graph shows 50% of the resulting runs, the green area extends this to show 75% of the resulting runs.

In *all* runs, the self-sustaining melting of permafrost is maintained in the model.

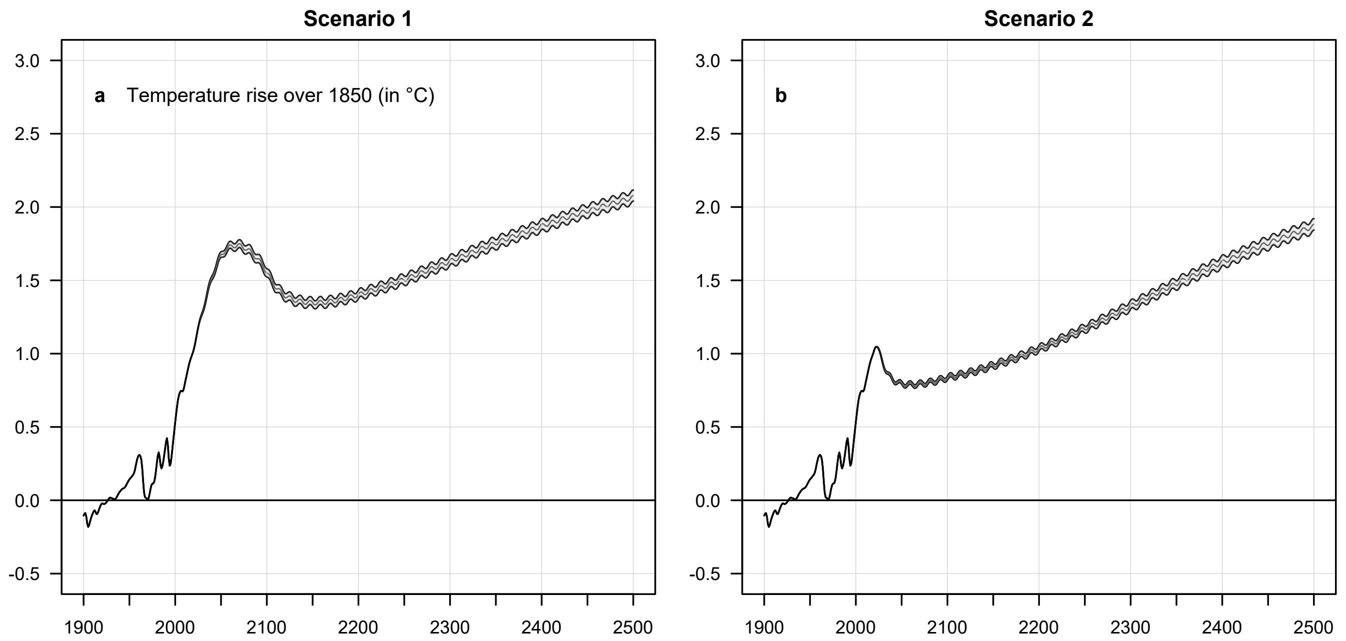

Supplement Figure 5: For scenario 1 (a) and 2 (b)

Sensitivity to change in the fraction of carbon that is converted (by bacteria) from CH_4 to CO_2 before it leaves the melting permafrost. In the base run of ESCIMO all C being released from thawing permafrost is released as CH_4. In these runs here we uniformly sample this fraction from 0% (all C released as CO_2) to 15% of all C released as CH_4 for 500 runs. The grey area in each graph shows 75% of the resulting runs.

In *all* runs, the self-sustaining melting of permafrost is maintained in the model.

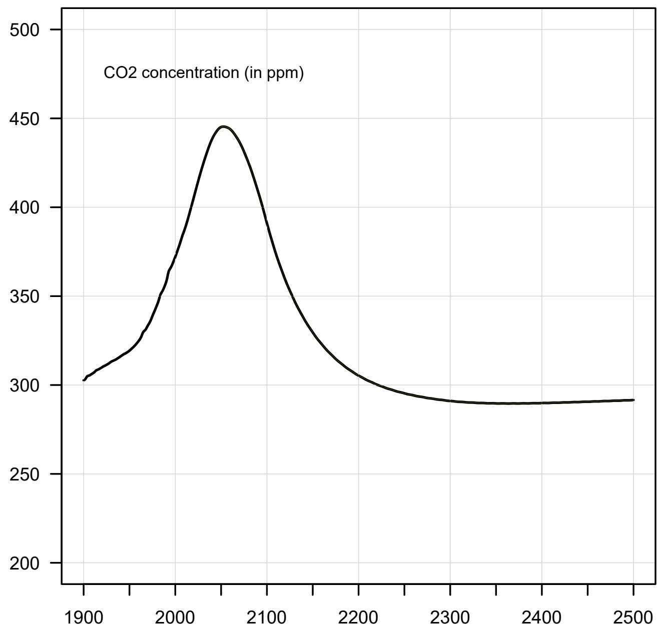

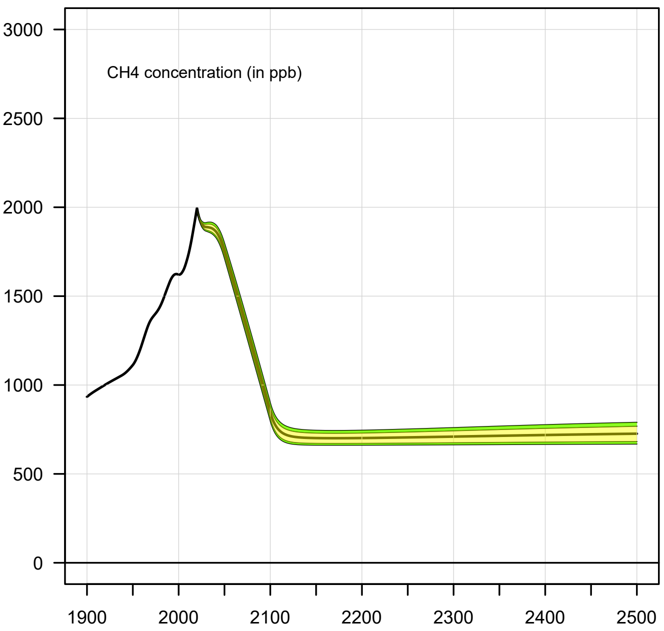

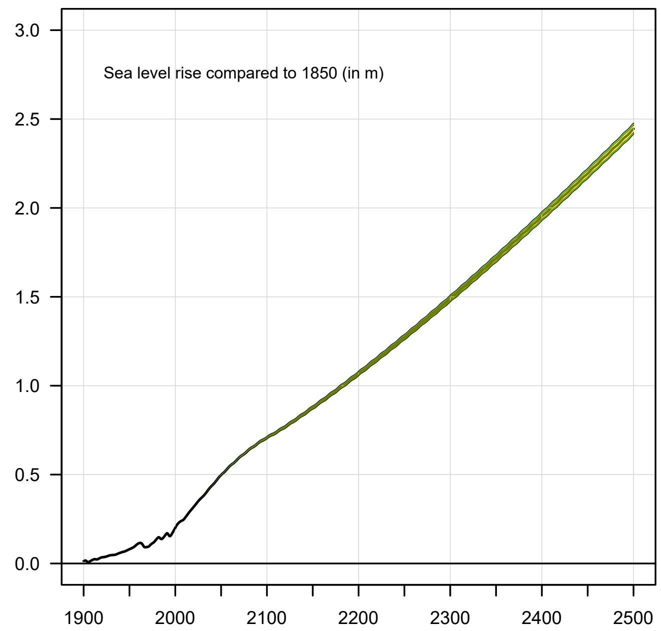

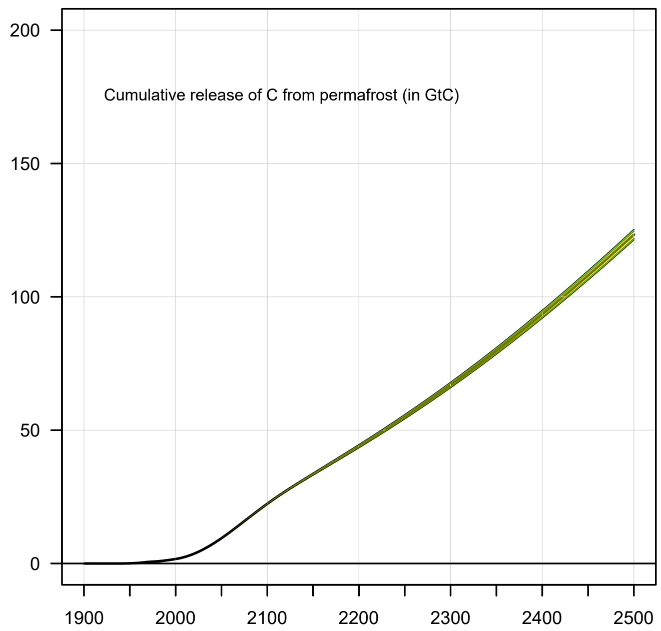

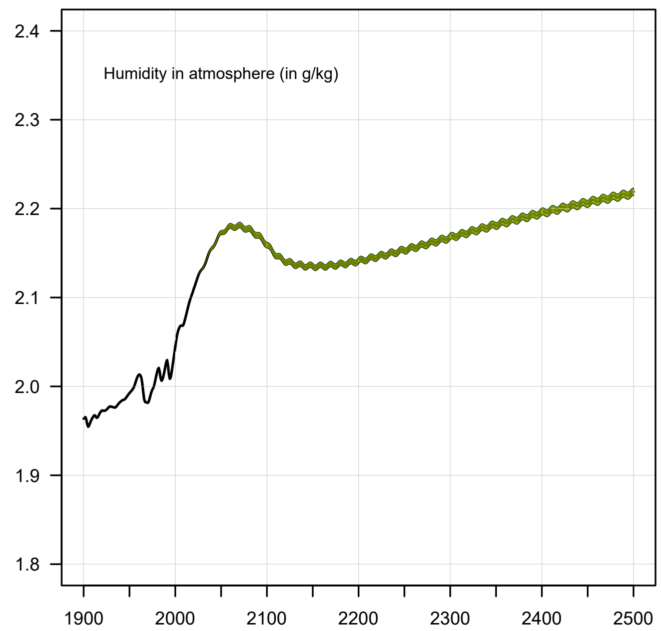

Supplement Figure 6: For scenario 1

Sensitivity to change in the fraction of carbon that is converted (by bacteria) from CH_4 to CO_2 before it leaves the melting permafrost. In the base run of ESCIMO all C being released from melting permafrost is released as CH_4. In these runs here we uniformly sample this fraction from 0% (all C released as CO_2) to 15% of all C released as CH_4 for 500 runs. The yellow area in each graph shows 50% of the resulting runs, the green area extends this to show 75% of the resulting runs.

In *all* runs, the self-sustaining melting of permafrost is maintained in the model.

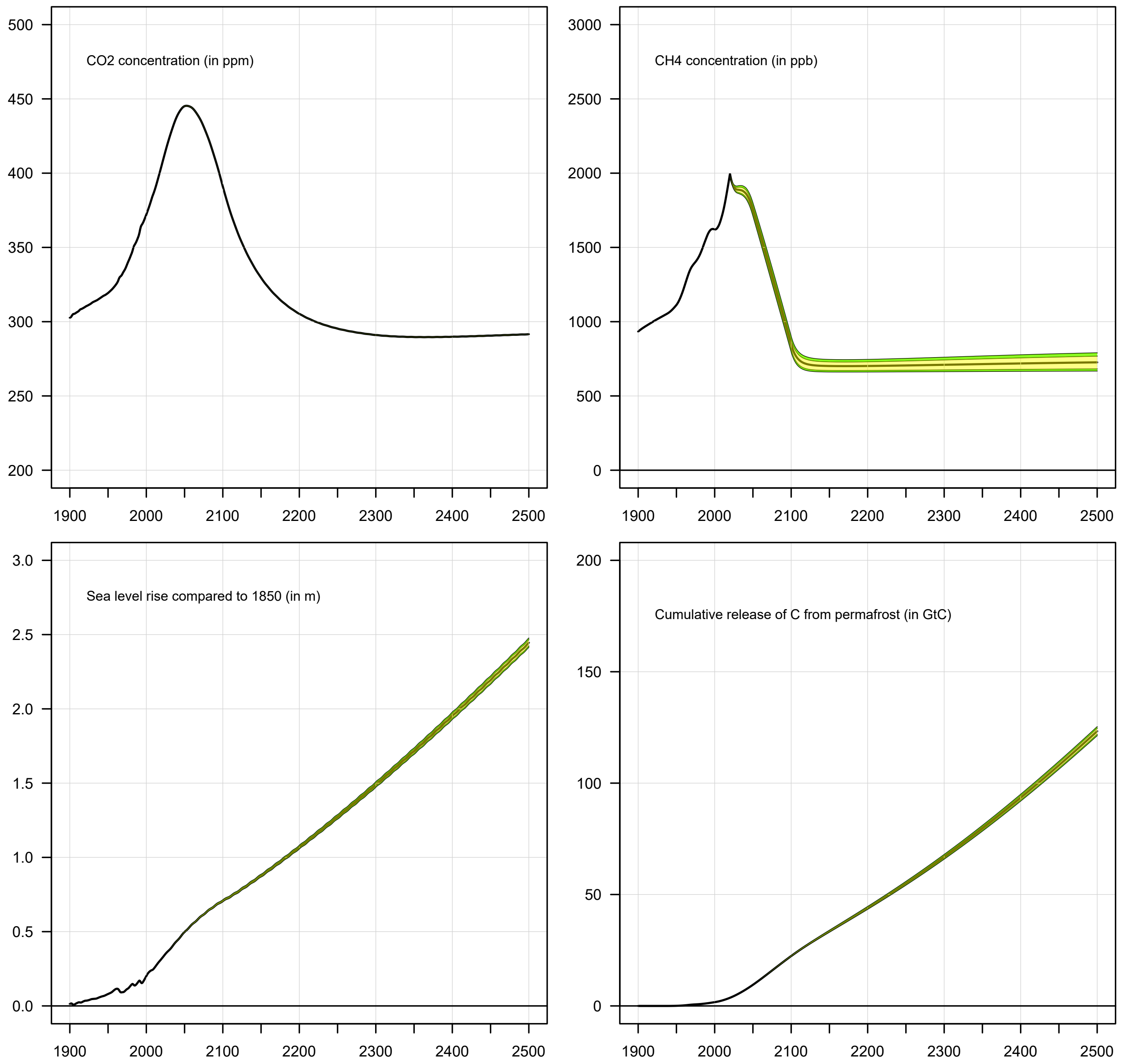

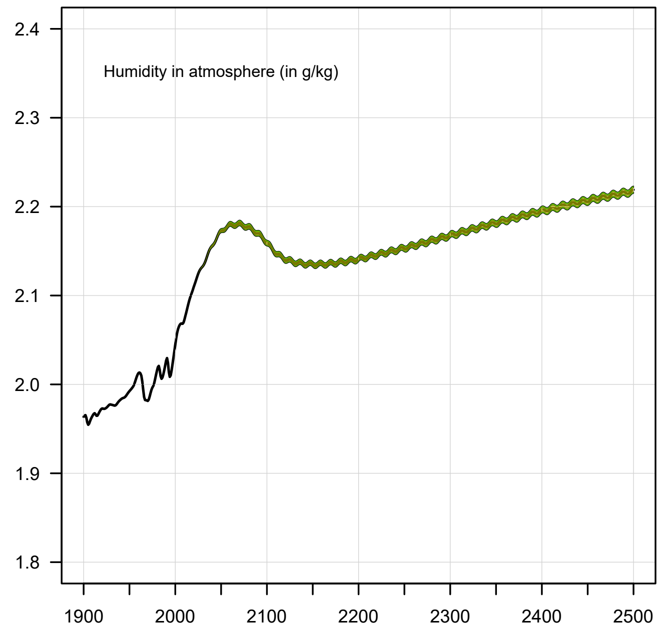

Supplement Figure 7: For scenario 2

Sensitivity to change in the fraction of carbon that is converted (by bacteria) from CH_4 to CO_2 before it leaves the melting permafrost. In the base run of ESCIMO all C being released from melting permafrost is released as CH_4. In these runs here we uniformly sample this fraction from 0% (all C released as CO_2) to 15% of all C released as CH_4 for 500 runs. The yellow area in each graph shows 50% of the resulting runs, the green area extends this to show 75% of the resulting runs.

In *all* runs, the self-sustaining melting of permafrost is maintained in the model.

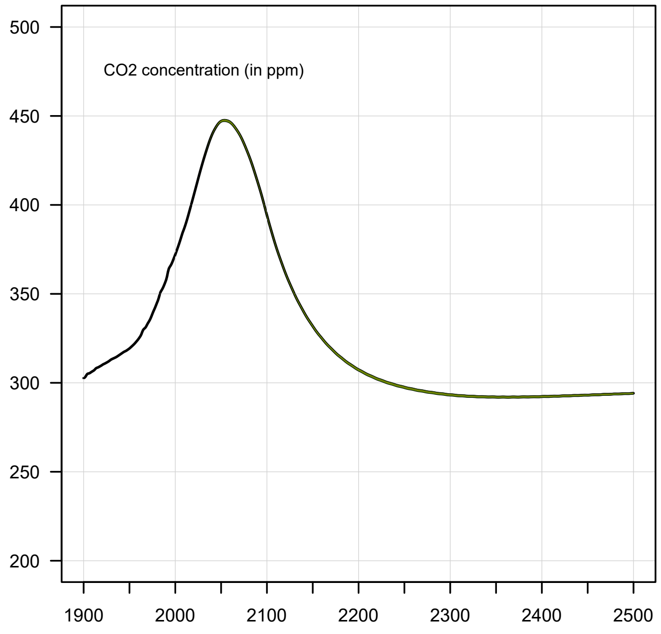

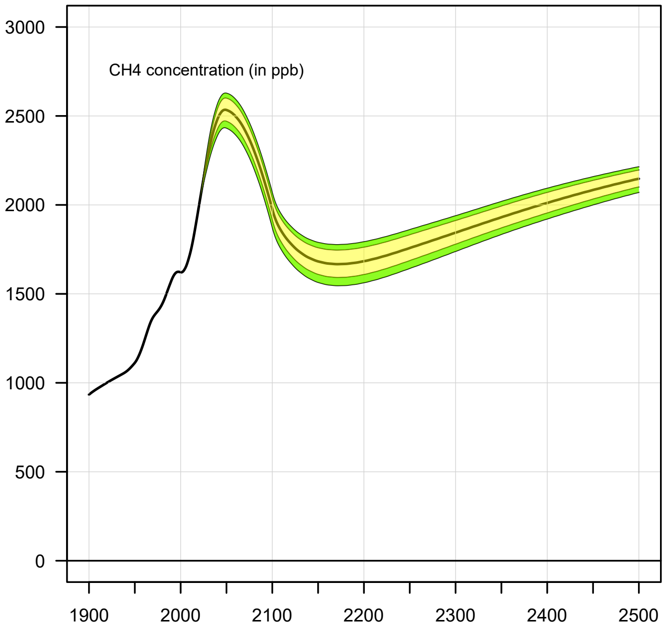

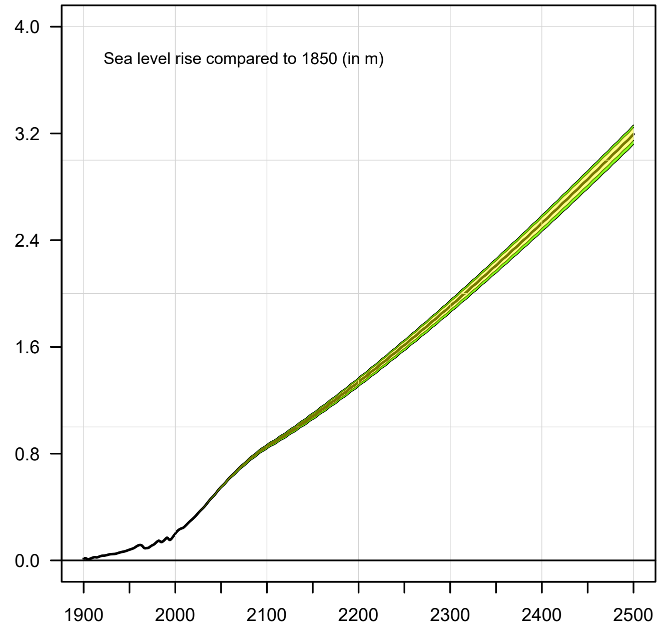

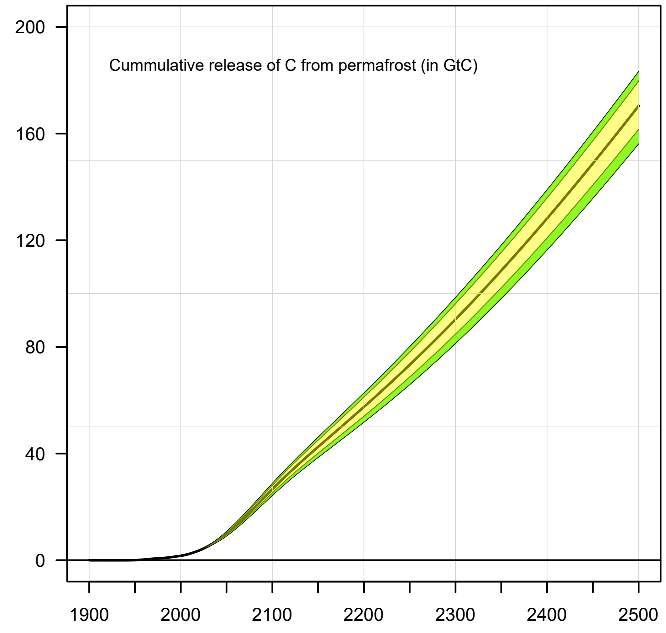

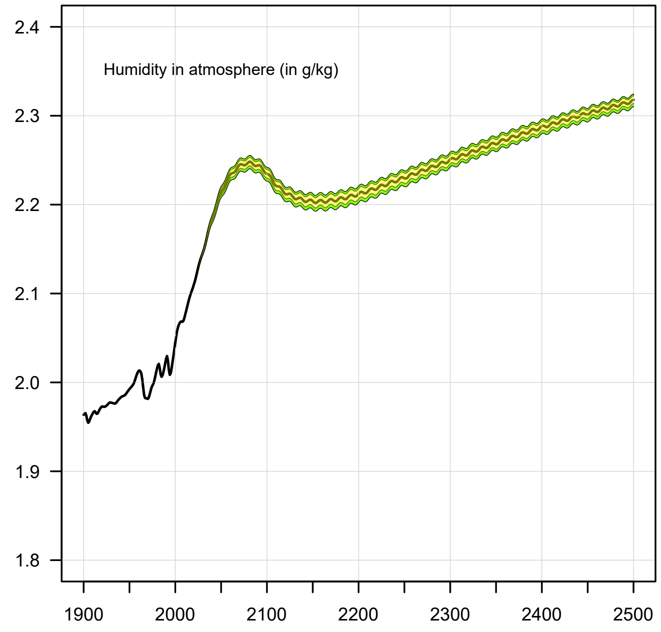

Supplement Figure 8: For scenario 1

Sensitivity to change in the slope of the rate of melting of the permafrost that results from a given temperature. We run 500 sensitivity runs where we pick values within plus or minus 10% of the base run slope (which is 1) from a uniform random distribution. The yellow area in each graph shows 50% of the resulting runs, the green area extends this to show 75% of the resulting runs.

In *all* runs, the self-sustaining melting of permafrost is maintained in the model.

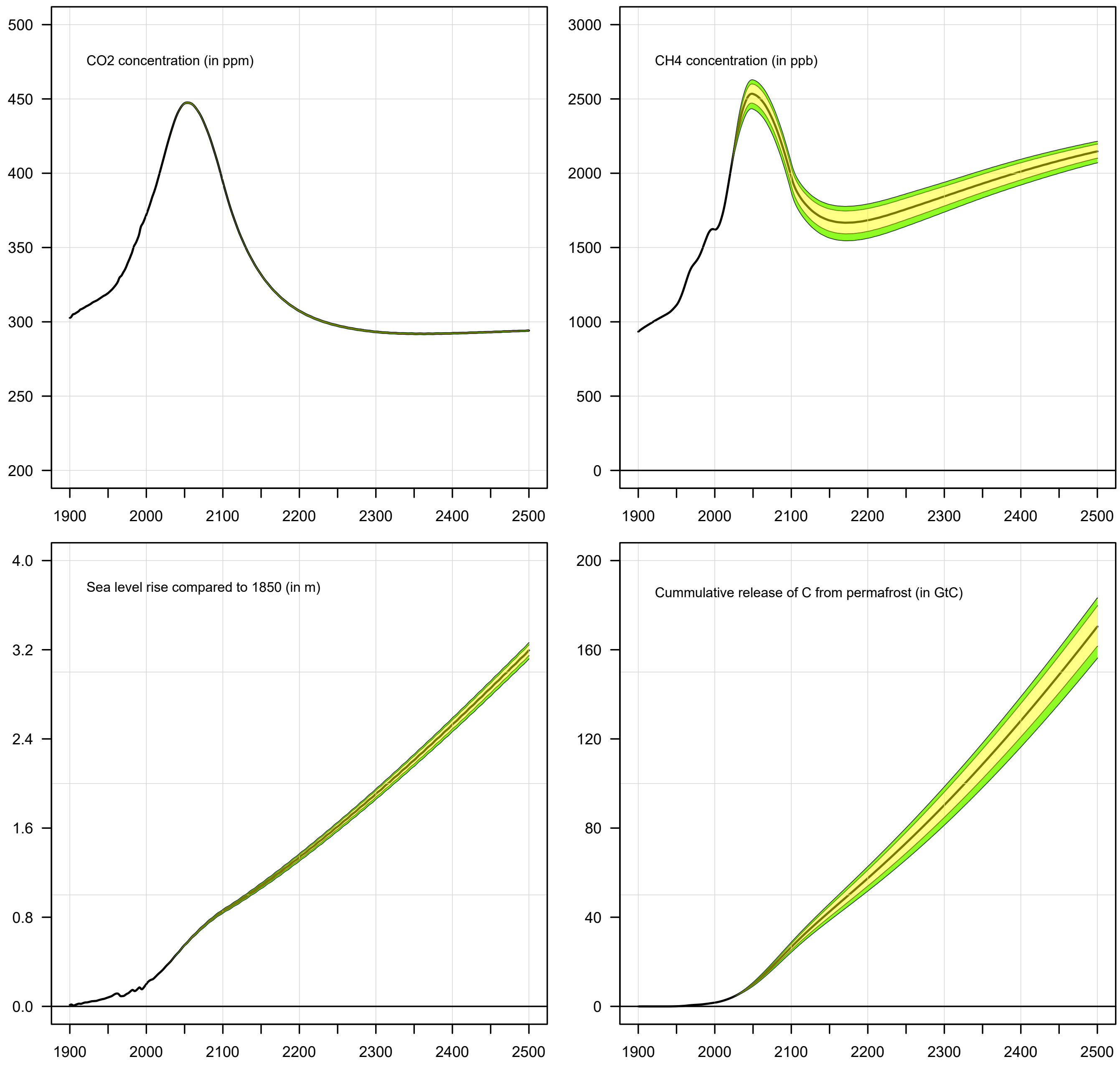

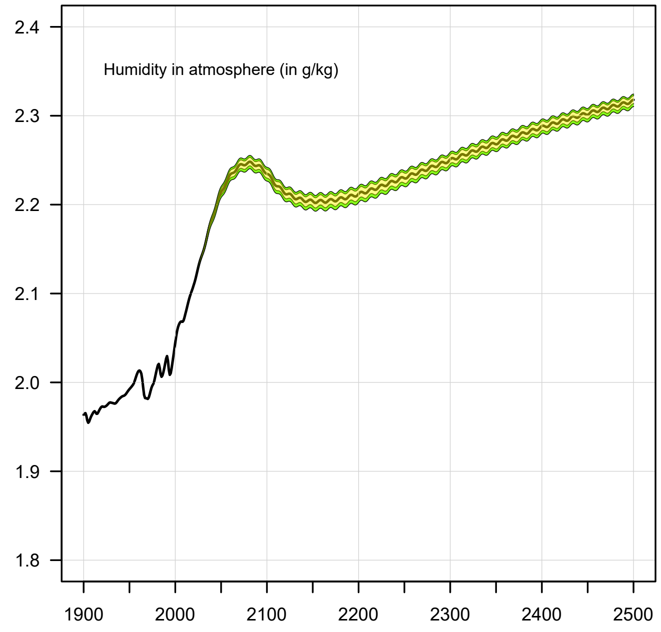

Supplement Figure 9: For scenario 2

Sensitivity to change in the slope of the rate of melting of the permafrost that results from a given temperature. We run 500 sensitivity runs where we pick values within plus or minus 10% of the base run slope (which is 1) from a uniform random distribution. The yellow area in each graph shows 50% of the resulting runs, the green area extends this to show 75% of the resulting runs.

In *all* runs, the self-sustaining melting of permafrost is maintained in the model.

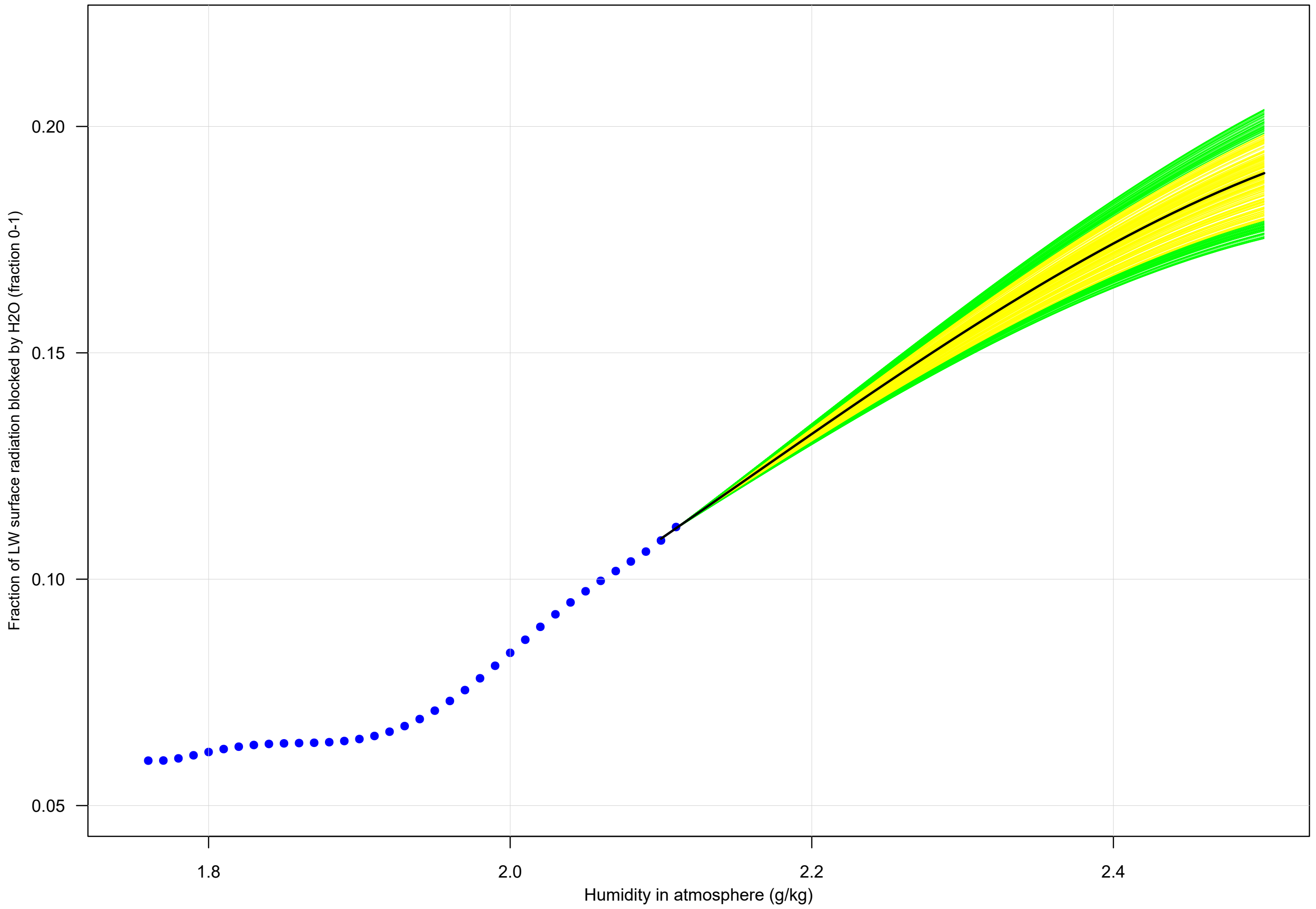

Supplement Figure 10

Sensitivity to change in the slope of the additional blocking of long wave surface radiation that results from additional water vapour in the atmosphere. Since the relationship is not linear, we could not change the slope by a fixed percentage to generate the sensitivity runs. Instead we created changes in the relationship as follows:

Blue dots represent the historical data we derived from calibrating the entire climate system to historical values of temperature, carbon and heat flows, albedo, etc.

The thick black line is the standard ESCIMO extension of history into in the region beyond what has been observed this far. The formula we use is a 3rd order polynomial $f(x) = - 0.2842 * humidity^3 + 1.8344 * humidity^2 - 3.7148 * humidity + 2.4523$, valid for the region of humidity from 2.1 to 2.5 g/kg.

The yellow lines show how the black line has been randomised for 50% of the sensitivity runs, the green lines show how the black line has been randomised for a further 75% of the sensitivity runs.

The relationship above reflects the lack of real world knowledge of the effect of water vapour, *not* a well-mixed GHG, on the blocking of heat transfer to space. Once we learn more about the real world relationship, we can incorporate the new knowledge into ESCIMO with relative ease.

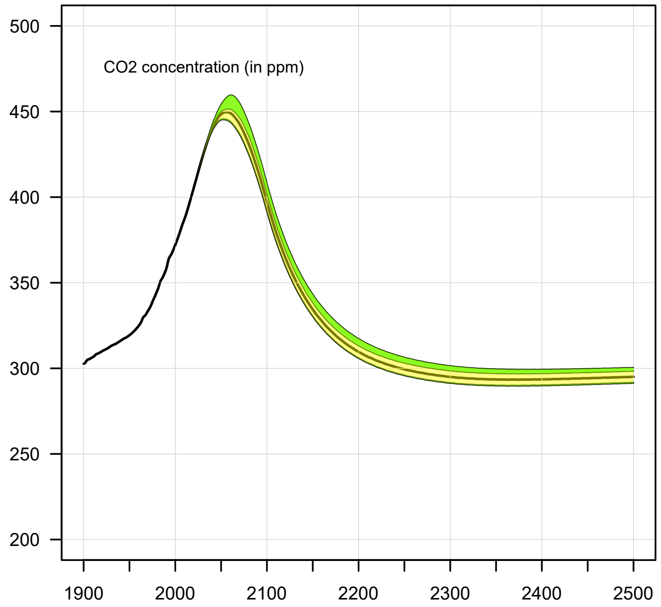

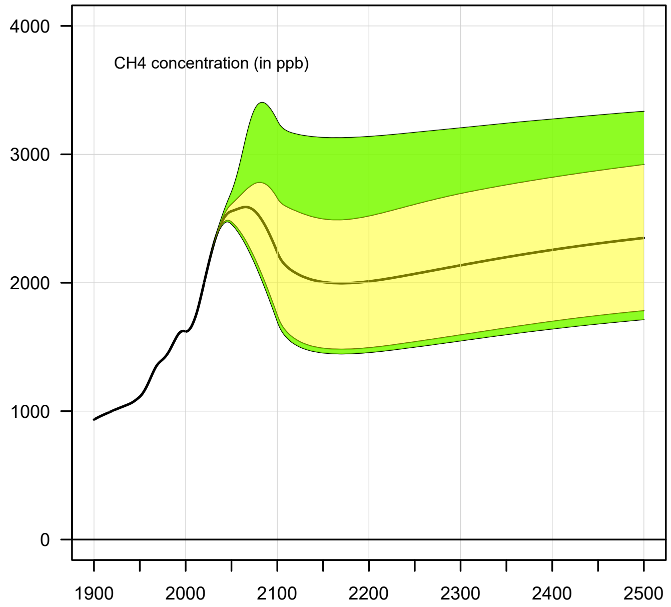

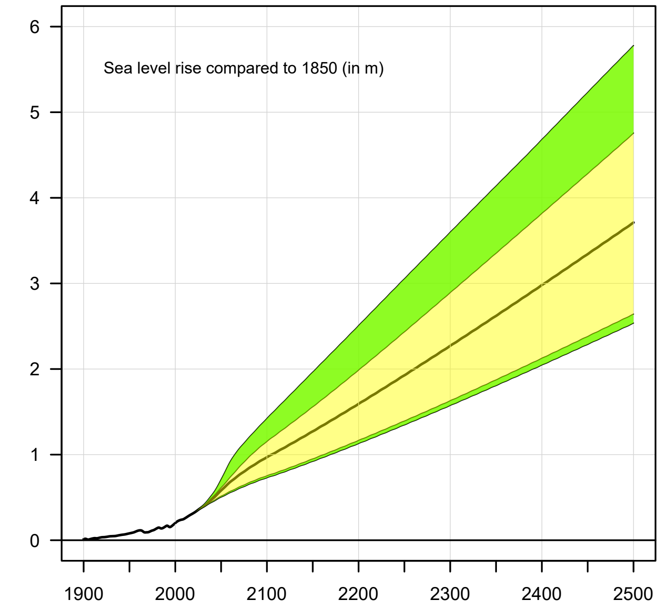

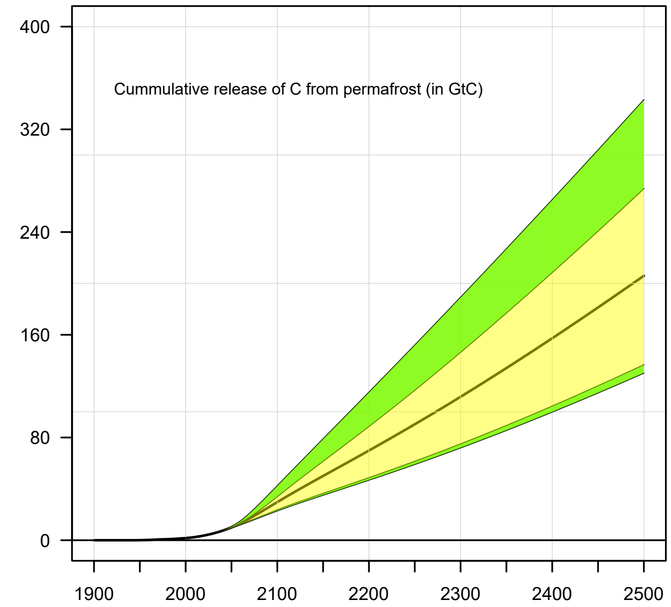

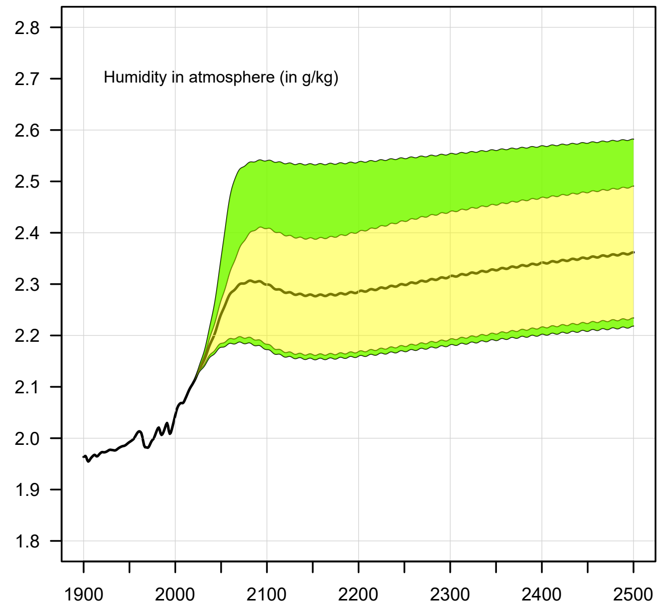

Supplement Figure 11: For scenario 1

Sensitivity to change in the slope of the additional blocking of outgoing radiation that results from additional water vapour in the atmosphere. How we change the slope of this relationship is detailed in Supplement Figure 10 on the previous page.

In *all* runs, the self-sustaining melting of permafrost is maintained in the model.

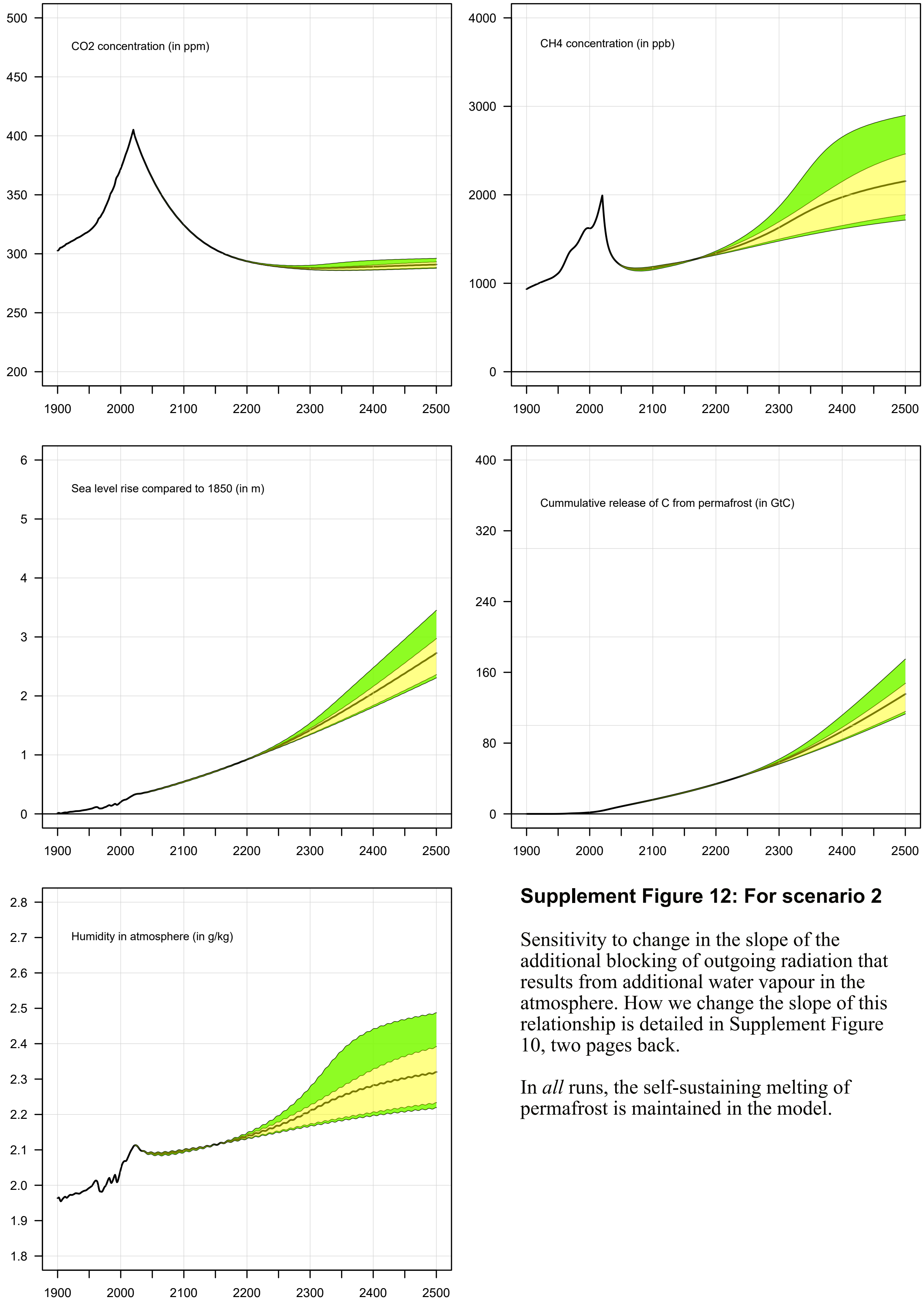

Supplement Figure 12: For scenario 2

Sensitivity to change in the slope of the additional blocking of outgoing radiation that results from additional water vapour in the atmosphere. How we change the slope of this relationship is detailed in Supplement Figure 10, two pages back.

In *all* runs, the self-sustaining melting of permafrost is maintained in the model.

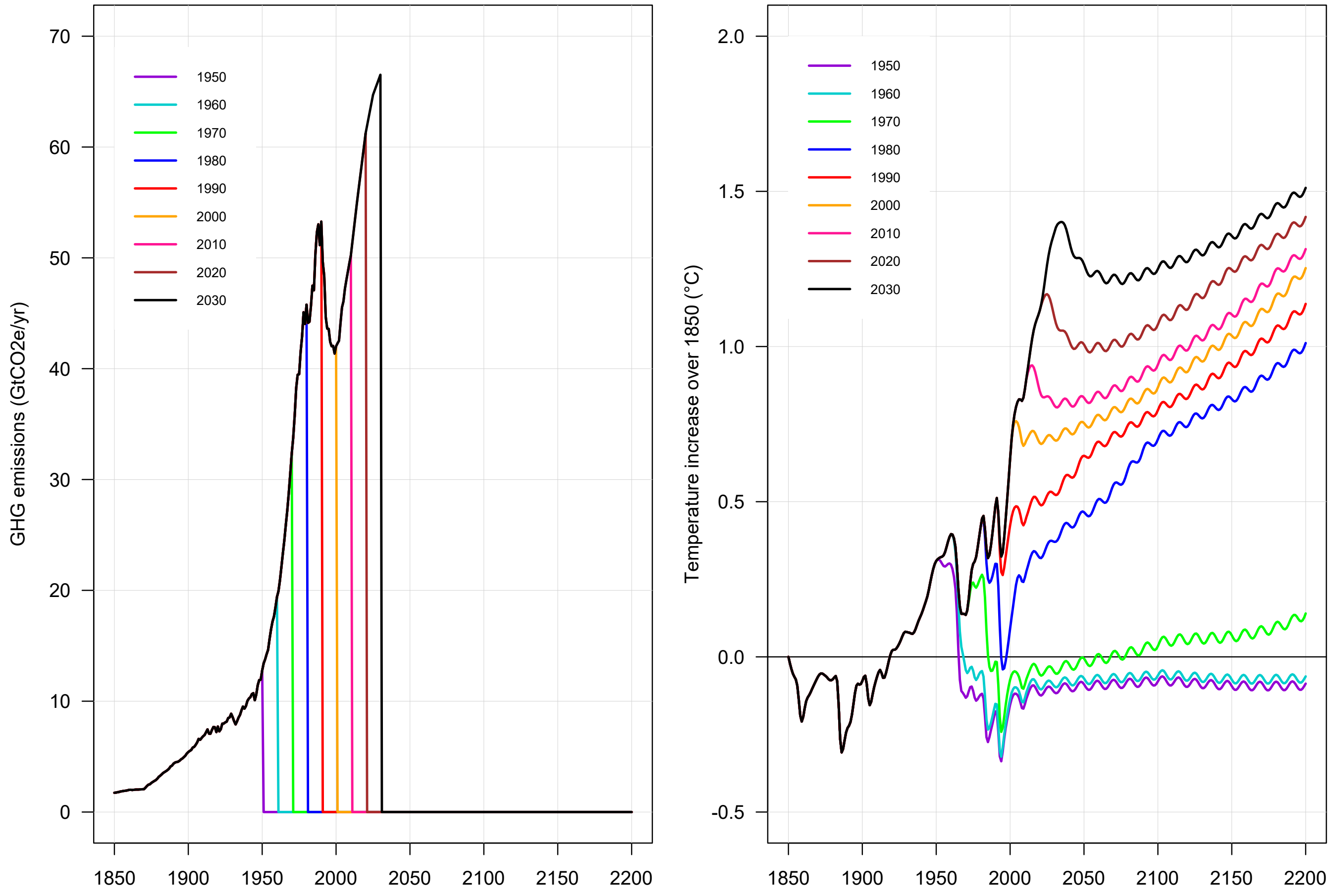

Supplement Figure 13

Another way to explore whether we have, in ESCIMO, passed the point-of-no-return in temperature rise is to run *counterfactual* experiments by cutting GHG emissions abruptly to zero at various points in the past.

The left panel above shows the cuts initiated at 10-year intervals from 1950 to 2030.

The right panel above shows the resulting global surface temperature difference to 1850 resulting from the various experiments. Sometime between 1960 and 1970 the trajectory takes on the characteristic self-sustaining pattern we see today.

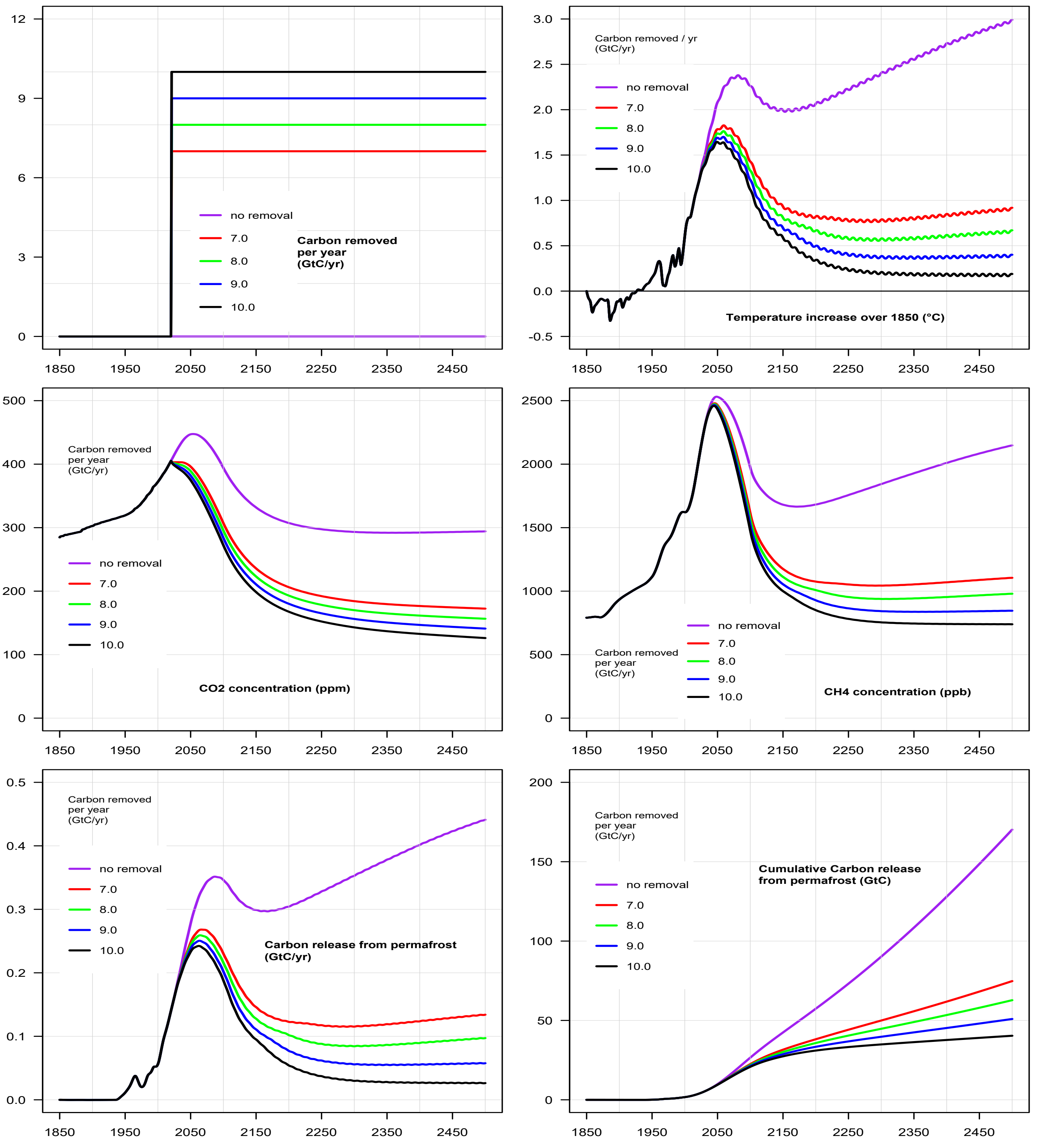

Supplement Figure 14 a (carbon in GtC)

We also tested, in ESCIMO, whether removing carbon from the atmosphere is sufficient to avoid self-sustaining temperature rise.

The top left panel above shows the removal experiments, under scenario 1.

The top right panel above shows the resulting global surface temperature difference to 1850 resulting from the various experiments. It is possible, in ESCIMO, to avoid self-sustaining temperature rise if 1) enough GHGes are removed annually, at least 10 GtC/yr (black curve), and 2) if this removal effort continues at least until 2500. The reason that removal has to continue for so long is that the combination of reduced albedo, CH_4 release and water vapour, elevated due to the higher temperature, tries to keep the temperature high. GHG removal thus has to overcompensate, which can be seen in the middle left panel where CO_2 concentration falls below pre-industrial levels past the year 2050.

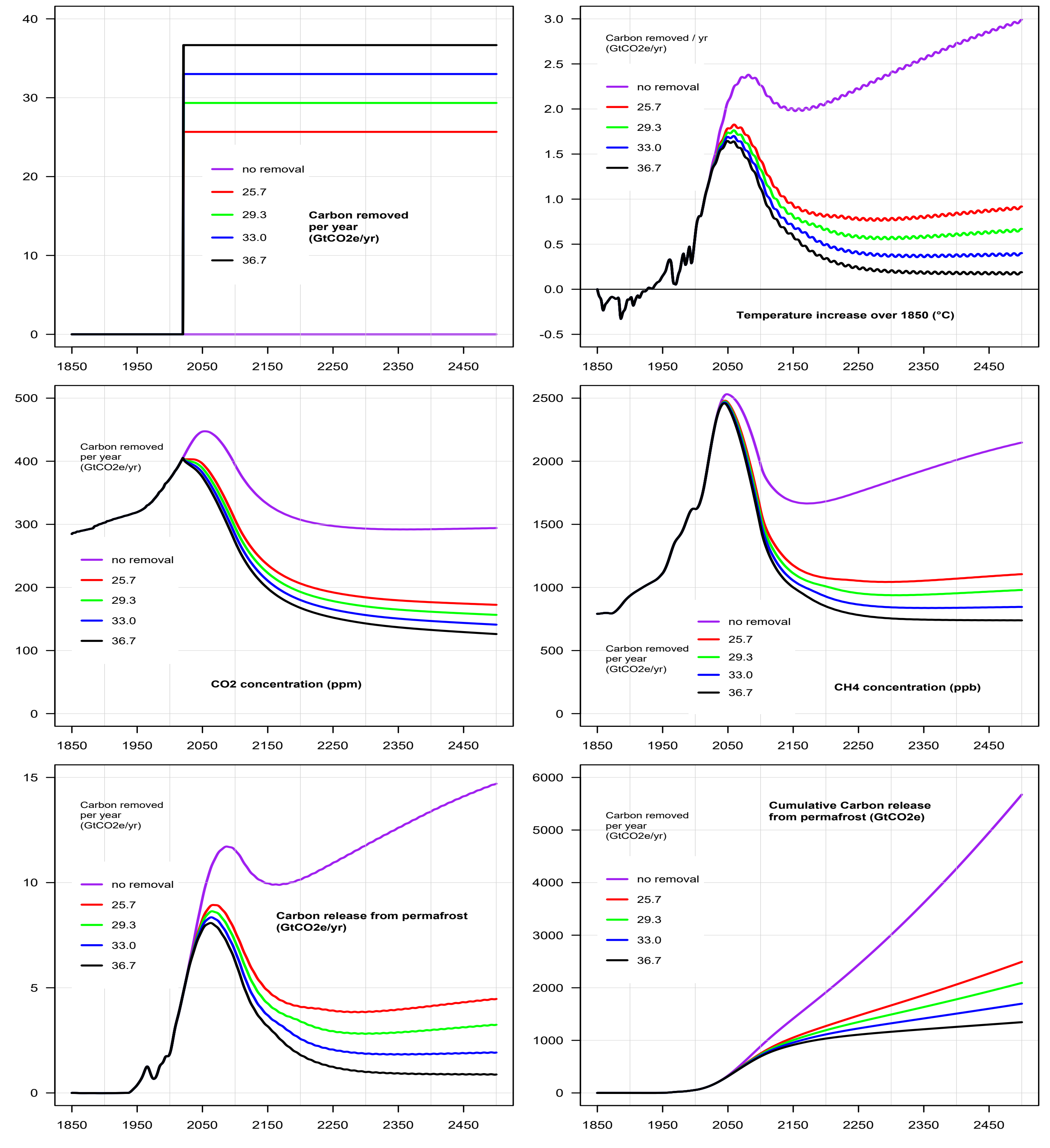

Supplement Figure 14 b (carbon in GtCO2e)

We also tested, in ESCIMO, whether removing carbon from the atmosphere is sufficient to avoid self-sustaining temperature rise. The top left panel above shows the removal experiments, under scenario 1. It is the same as in Supplement Figure 14 a, but carbon is shown in $GtCO_2e$. ESCIMO runs in GtC throughout. Thus, when we calculate the effect of GHG molecules once they are in the atmosphere, we use the instantaneous effect. Since people are used to seeing summaries of all GHGs in $GtCO_2e$, we use, for display purposes when we show these summaries, the 100 year global warming potential method to convert gases to CO_2e.

The top right panel above shows the resulting global surface temperature difference to 1850 resulting from the various experiments. It is possible, in ESCIMO, to avoid self-sustaining temperature rise if 1) enough GHGes are removed annually, at least 33 $GtCO_2e$/yr (black curve), and 2) if this removal effort continues at least until 2500. The reason that removal has to continue for so long is that the combination of reduced albedo, CH_4 release and water vapour, elevated due to the higher temperature, tries to keep the temperature high. GHG removal thus has to overcompensate, which can be seen in the middle left panel where CO_2 concentration falls below pre-industrial levels past the year 2050.

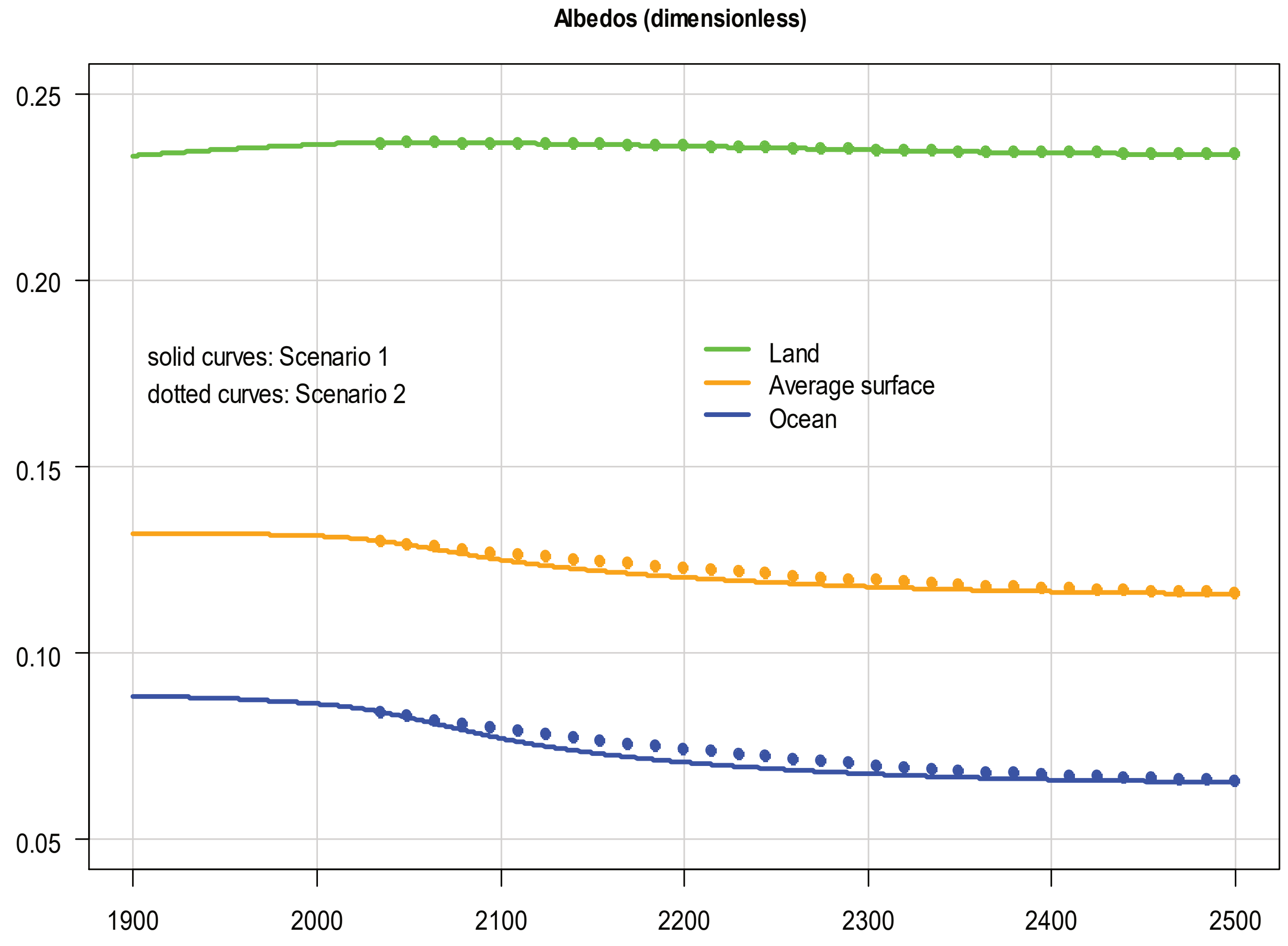

Supplement Figure 15: Surface albedos in ESCIMO.

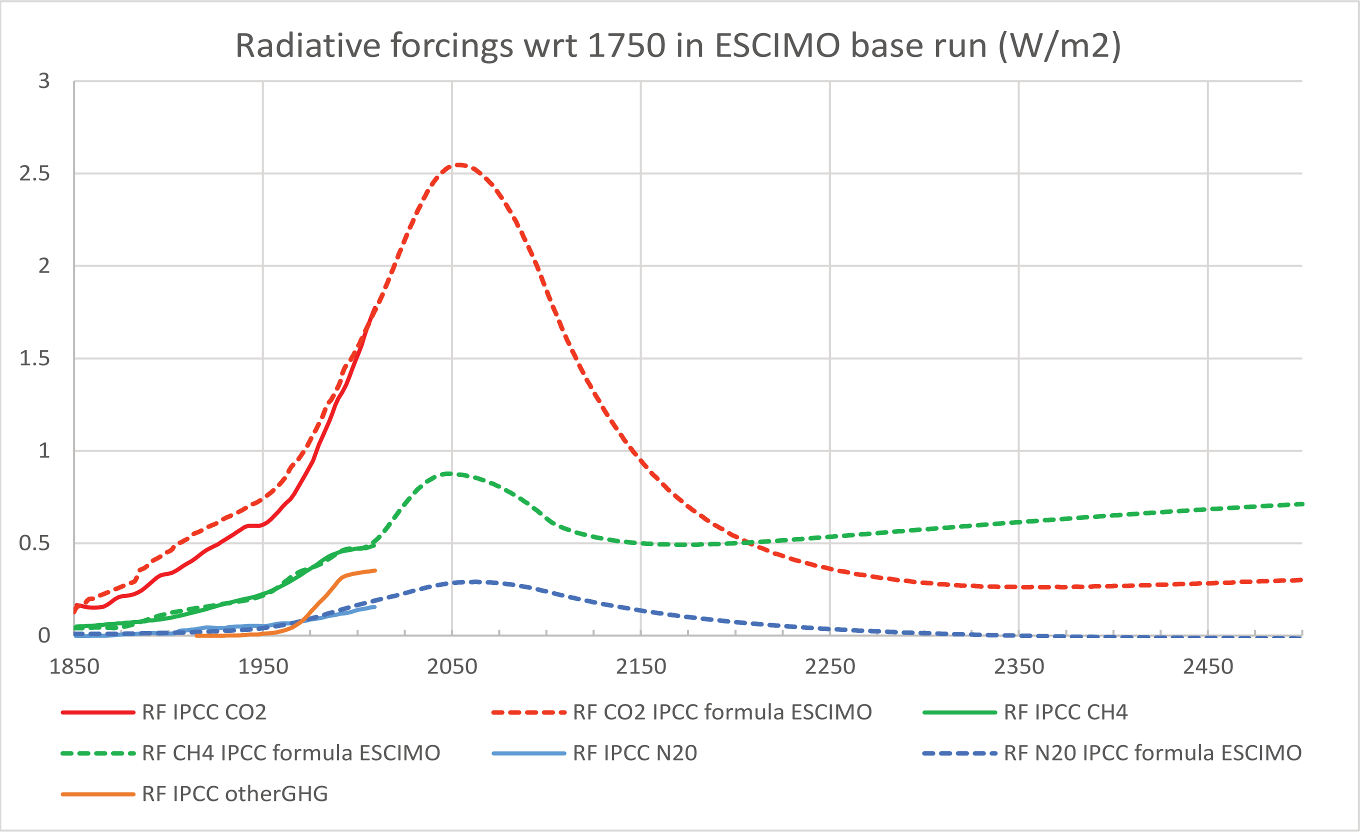

Supplement Figure 16: Radiative forcings over time calculated from IPCC formulas.

We computed the effect on radiative forcing for CO_2, CH_4 and N_2O we used the IPCC formulas. For all three GHG we use model generated concentrations for our ESCIMO calculations. For the IPCC calculations we replicate their Figure 8.6 (a) in Myhre, G., D. Shindell, F.-M. Bréon, W. Collins, J. Fuglestvedt, J. Huang, D. Koch, J.-F. Lamarque, D. Lee, B. Mendoza, T. Nakajima, A. Robock, G. Stephens, T. Takemura and H. Zhang, 2013: Anthropogenic and Natural Radiative Forcing Supplementary Material. In: Climate Change 2013: The Physical Science Basis. Contribution of Working Group I to the Fifth Assessment Report of the Intergovernmental Panel on Climate Change [Stocker, T.F., D. Qin, G.-K. Plattner, M. Tignor, S.K. Allen, J. Boschung, A. Nauels, Y. Xia, V. Bex and P.M. Midgley (eds.)]. Available from www.climatechange2013.org and www.ipcc.ch.
